# Social Media as a Tool for Public Health: Reviewing the Use of Social Media for Disease Surveillance, Behavioral Change, and Misinformation Control

**DOI:** 10.7759/cureus.111303

**Published:** 2026-06-22

**Authors:** Victor C Okebalama, Onyebuchukwu Eke, Cyprian Okoronkwo, Odutola Odugbemi, Joshua Odeyinka, Cyril Akwuruoha, David Akwuruoha, Christian Ugochukwu, Osinaike Abiodun, Ngozi Adefala, John olawoye, Kehinde Ogunlesi, Chidinma Halliday, Adaeze Alinnor, Oluwatobi Aremu, Charles Odebiyi

**Affiliations:** 1 Anatomic Pathology and Forensic Medicine, Babcock University Teaching Hospital, Ilishan-Remo, NGA; 2 Oncology/Toxicology, Nigeria Police Directorate of Medical Services, Abuja, NGA; 3 Pharmacology and Therapeutics, Ebonyi State University, Abakaliki, NGA; 4 Internal Medicine, King Fahad Specialist Hospital, Tabuk, SAU; 5 Obstetrics and Gynaecology, Babcock University Teaching Hospital, Ilishan-Remo, NGA; 6 Surgery, Babcock University Teaching Hospital, Ilishan-Remo, NGA; 7 Obstetrics and Gynaecology, Rivers State University Teaching Hospital, Port Harcourt, NGA; 8 Internal Medicine, Benjamin S. Carson (Snr) College of Health and Medical Sciences, Babcock University, Ilishan-Remo, NGA; 9 Paediatrics, Babcock University Teaching Hospital, Ilishan-Remo, NGA; 10 Community Medicine, Babcock University Teaching Hospital, Ilishan-Remo, NGA; 11 Internal Medicine, Federal Medical Centre, Umuahia, NGA; 12 Internal Medicine, Babcock University Teaching Hospital, Ilishan-Remo, NGA

**Keywords:** disease surveillance, facebook, influence and behavior, public communication, public health, public health promotion, social media, strategies, twitter, whatsapp

## Abstract

Social media has transformed public health communication from a one-sided approach to a more interactive model, facilitating discussions on symptoms, treatments, and personal experiences among patients and caregivers, often without health professionals' involvement. Public health agencies recognised social media's potential for cost-effective, rapid outreach and real-time feedback, influencing health perceptions and behaviors. However, this platform also risks amplifying misinformation and conflicting narratives. Beyond communication, social media supports disease surveillance, influences health behaviors, and disseminates critical information during emergencies, improving responses while requiring careful risk communication to prevent confusion. The need for a review is emphasised to analyse social media's role in health promotion, risks, and ethical integration into health systems, focusing on its impact, methods utilised, and balancing transparency with misinformation management. This review aims to create a literature-informed narrative that guides future research and public health communication strategies.

## Introduction and background

Social media has transformed health information dissemination from a one-way communication model to an interactive, participatory approach [1,2]. In the early 2010s, personal networking platforms became venues for extensive health discussions, allowing patients and caregivers to seek advice and share experiences without direct health professional involvement [1,2]. This shift democratised health information, moving it beyond clinical settings [1,2]. At the same time, public health actors began to recognise social media's potential for rapid, cost-effective outreach, enabling targeted messaging and real-time feedback [1,2]. However, while social media can amplify accurate information, it also presents challenges by spreading misinformation and conflicting narratives [1,3]. This has necessitated new frameworks for public health communication and engagement [1-3].

Social media has evolved beyond basic communication to play a critical role in public health by supporting disease surveillance, influencing health behaviors, and disseminating information during emergencies and routine situations [4,5]. User-generated content across platforms can be analysed to detect early disease signals and monitor community responses, particularly in areas with weak reporting systems [4,5]. At the behavioural level, these platforms have been used to design and deliver targeted health promotion campaigns, often using tailored messages, visual materials, and interactive formats that appeal to specific age groups or cultural contexts [5,6]. Social media facilitates targeted health promotion campaigns designed for specific demographics and has demonstrated effectiveness in improving health awareness and behaviors, such as vaccination uptake [5,6]. Furthermore, agencies use these platforms to share timely updates on health threats and responses, employing varied formats to enhance understanding [4,5]. This highlights the multi-functional nature of social media in public health, serving as both a monitoring tool and an intervention mechanism [4-6].

The growing role of social media in public health necessitates a systematic review to assess its applications, impacts, and ethical integration into health systems [7,8]. Current findings indicate that while social media aids in health promotion, risk communication, and surveillance, it also presents challenges like misinformation and privacy issues [7,8]. Despite limited long-term evidence, public health entities are increasingly adopting these platforms, risking uncoordinated strategies that may harm public trust and reduce the effectiveness of public health efforts [7-9].

This narrative review focuses on peer-reviewed literature examining social media platforms and their impact on public health outcomes. It explores how platforms like Twitter, Facebook, WhatsApp, and video channels are utilised, the analytical methods applied by researchers, and institutional responses to new opportunities and challenges. Ethical considerations such as privacy, digital divides, and representation are also discussed. This narrative review emphasises the need to balance enhancing transparency and engagement with mitigating the spread of misinformation, aiming to inform future research, policy, and public health communication strategies based on empirical evidence.

## Review

Methods

A comprehensive literature evaluation was conducted over a duration of 16 months (January 1st, 2025, to April 30th, 2026) using the databases Google Scholar, the Cochrane Library, PubMed, ScienceDirect, and Scopus. Keywords like "disease surveillance", "public health", "social media", "Facebook", "WhatsApp", "health platforms", "public communication", "strategies", "influence", "scope", "spread", "reshaped", and "design" were combined in tandem with Boolean operators (AND, OR) to find pertinent recent academic publications authored in the English language and to focus and refine the results. The combined set of search terms for PubMed is (("social media"[All Fields] OR "public health"[All Fields] (("social media"[All Fields] AND "public health"[All Fields] ((social media"[All Fields] OR "disease surveillance"[All Fields] (("social media"[All Fields] AND "disease surveillance"[All Fields] (("Facebook"[All Fields] OR "public health"[All Fields] (("Facebook"[All Fields] AND "public health"[All Fields] (("WhatsApp"[All Fields] OR "public health"[All Fields] (("WhatsApp "[All Fields] AND "public health"[All Fields] (("social media"[All Fields] OR "health influence"[All Fields] (("social media"[All Fields] AND "health influence"[All Fields] (("social media"[All Fields] OR "health behaviour" [All Fields] (("social media"[All Fields] AND "health behaviour"[All Fields] (("social media"[All Fields] OR "health communication" [All Fields] (("social media" [All Fields] AND "health communication" [All Fields] (("social media" [All Fields] OR "health information" [All Fields] (("social media" [All Fields] AND "health information" [All Fields] (("social media" [All Fields] OR "health misinformation" [All Fields] (("social media"[All Fields] AND "health misinformation"[All Fields] (("Twitter"[All Fields] OR "public health" [All Fields] (("Twitter" [All Fields] AND "public health" [All Fields] (("social media" [All Fields] OR "health promotion" [All Fields] (("social media" [All Fields] AND "health promotion" [All Fields] (("social media" [All Fields] OR "ethical considerations" [All Fields]) (("social media" [All Fields] AND "ethical considerations" [All Fields]. Systematic reviews, scholarly consensus publications, landmark research investigations, and contemporary outstanding cohort research studies pertinent to social media as a tool for public health, including disease surveillance, behavioural change, and misinformation control of its use, were prioritised. Also, bibliographic lists from some chosen papers were manually screened to find further references.

Furthermore, no systematic meta-analysis or quantitative evidence synthesis was carried out because the current research article was intended to provide a thorough narrative assessment of social media as a tool for public health.

The evolution of social media platforms and their integration into public health systems

Early Development of Social Media Platforms and Initial Public Health Uses

In the early 2000s, before the rise of today’s mainstream social media platforms, people began using blogs and online forums as informal spaces to discuss health‑related topics [1,2]. These early digital environments allowed patients, caregivers, and lay users to share personal experiences with diseases, treatments, and side effects, often in the absence of immediate professional supervision [1,2]. Over time, these platforms became repositories of anecdotal knowledge, where individuals sought reassurance, compared symptoms, and exchanged practical advice about managing chronic conditions or navigating healthcare systems [1,2]. Although the information shared was often unregulated and not formally validated, the interactive nature of blogs and forums made them distinct from one‑way sources like printed leaflets or basic health websites [1,2]. As internet access expanded, these early platforms laid the groundwork for what would later become more structured social media ecosystems [1,2]. Users began to form communities around particular conditions, such as diabetes, mental health, or rare diseases, where shared narratives helped normalise experiences and reduce feelings of isolation [1,2]. For public health practice, this development was significant because it showed that health information was no longer confined to clinical settings or official channels; instead, it was being generated and circulated in decentralised, user‑driven spaces [1,2]. Later work has highlighted how such early informal channels set the stage for the broader integration of social media into public health communication, as authorities gradually recognised the need to engage with, rather than ignore, these emerging digital forums [1,2,10].

The move from Web 1.0, where most users were passive consumers of static web pages, to Web 2.0 transformed how people interacted with online content, including health information [3,7]. Web 2.0 introduced features such as commenting, sharing, and user‑generated content, which allowed individuals to participate in discussions rather than simply read pre‑prepared information [3,7]. In public health, this meant that messages about vaccination, healthy lifestyles, or disease prevention were no longer limited to one‑way broadcasts; they could be discussed, questioned, and adapted in real time by users [3,7]. The interactive nature of Web 2.0 also enabled users to build networks, join groups, and follow experts, which created new pathways through which health‑related knowledge could spread and be reshaped by peers [3,7]. This shift had important implications for how public health communication evolved [3,7]. Early reviews emphasise that the transition from static information delivery to participatory platforms expanded the potential for dialogue, feedback, and co‑creation of health messages [3,7]. As social media tools built on Web 2.0 principles became more widespread, agencies began to see them not only as channels for outreach but also as spaces where public sentiment, concerns, and behaviours could be monitored and addressed [3,7]. Later analyses of health‑communication frameworks have shown that this move from monologue to dialogue required new skills in listening, moderating, and responding, rather than merely producing content [3,7]. In this way, the transition from Web 1.0 to Web 2.0 marked a structural change in the relationship between public health institutions and the public, setting the stage for deeper integration of social media into practice [3,7,11].

In the 2010s, public health agencies began experimenting with social media as part of awareness‑raising campaigns, moving beyond traditional print and broadcast methods [5,12]. These early uses were often pilot‑level or ad hoc, but they demonstrated that platforms could reach large audiences quickly and at relatively low cost, especially for time‑sensitive health messages [5,12]. Agencies used social media to promote vaccination, healthy lifestyles, and disease prevention, tailoring content to different age groups and cultural contexts through short posts, infographics, and videos [5,12]. By tracking engagement metrics such as likes, shares, and comments, institutions could also gain insights into which messages resonated most with the public and refine their strategies accordingly [5,12]. At the same time, these early efforts also highlighted how social media could support two‑way communication, allowing agencies to receive feedback and clarify misunderstandings in real time [5,12]. Systematic reviews of public‑health responses during the COVID‑19 pandemic have shown that many agencies used these platforms to share updates on case trends, preventive measures, and policy changes, often in multiple languages and formats [4,5]. These experiences built on earlier work that documented how social media could be used to reinforce routine health‑promotion activities, such as campaigns against smoking or for physical activity [5,12]. Taken together, the initial use of social media for awareness campaigns marked a shift from treating digital channels as optional add-ons to treating them as integral components of public-health communication portfolios [4,5,12].

During several infectious disease outbreaks, public health actors began to use social media as a channel for sharing timely information and coordinating communication with the public [6,13]. In these early cases, platforms were used to announce suspected cases, provide guidance on prevention, and correct local rumours that could undermine control efforts [6,13]. Because information on social media could spread very quickly, agencies found that they had to respond rapidly to emerging questions and misinformation, even when formal investigation or laboratory confirmation was still underway [6,13]. In some settings, particularly where formal reporting systems were weak or fragmented, social‑media‑based discussions served as informal early‑warning signals of disease activity, even though they were not always systematically integrated into official surveillance [6,13]. One notable example comes from India, where WhatsApp groups were used informally to share suspected disease reports and coordinate community‑level responses, illustrating how platforms could become part of local outbreak communication practices [14]. Such experiences revealed both the potential and the risks of relying on social media during outbreaks: while it could speed up information flow and facilitate coordination, it could also amplify fear, conflicting narratives, and false claims that competed with official guidance [13,14]. Working papers evaluating the use of social media in public health surveillance have shown that early outbreak communication often lacked clear protocols for verification, moderation, and risk communication, leading to inconsistent quality of information [6,13]. Over time, these early case examples helped shape more structured approaches, in which social media was treated as a complementary tool rather than a stand‑alone system for outbreak communication [6,13,14].

Despite the promise of social media for public health, its early adoption faced several structural and cultural barriers [9,15]. Many institutions lacked the digital infrastructure, clear guidelines, and trained personnel needed to use these platforms safely and consistently [9,15]. In some cases, staff were hesitant to engage with social media because of concerns about privacy, data security, and the risk of misinformation or public criticism [8,15]. This reluctance was reinforced by a lack of formal policies on how to post, respond to comments, or manage online crises, which led to inconsistent practices across departments and agencies [8,15]. At the same time, there was limited institutional trust in the reliability of social‑media‑generated information, especially when it came from informal sources or unverified users [8,9]. Some professionals questioned whether data from platforms could be validated or integrated into formal decision‑making processes, which slowed efforts to embed social media into routine public‑health workflows [8,15]. Review‑style work on the role of social media in health has highlighted the need for better quality‑control mechanisms, training, and governance frameworks if these tools were to move from experimental use to stable, system‑level integration [8,9]. In low‑resource settings, additional constraints such as unreliable internet access, limited digital literacy, and uneven device ownership further restricted the reach and effectiveness of early social‑media‑based interventions [9,15]. Taken together, these limitations showed that technological availability was not enough; institutional culture, infrastructure, and trust had to evolve in parallel if social media were to become a dependable component of public health systems [8,9,15].

Institutional Adoption of Social Media by Public Health Organisations

Over time, public health agencies began to treat social media as a core element of their official communication strategies, rather than as an external or experimental tool [4,5]. Many organisations developed standard operating procedures for posting, responding to comments, and monitoring online discussions related to priority health issues [4,5]. These strategies often linked social‑media activities to broader public‑health goals, such as improving vaccination rates, promoting healthy lifestyles, or supporting outbreak preparedness, ensuring that digital messaging was aligned with offline programmes [4,5]. By embedding social media into strategic planning, agencies shifted from ad hoc posting to a more coordinated use of these platforms across different levels of the health system [4,5]. In parallel, global and regional guidance has emphasised the importance of integrating social media into formal emergency‑communication frameworks, especially during large‑scale health events [4,5]. Reviews of public‑health responses before and during the COVID‑19 pandemic have shown that agencies that already had social‑media communication plans in place were better able to share timely updates, manage public expectations, and correct misinformation [4,5]. In addition, these organisations increasingly used analytics to assess which messages generated the most engagement and which audiences were hardest to reach, allowing for more targeted and adaptive communication [5,16]. As a result, social media became less of a peripheral channel and more of a structured component of public health communication infrastructure [4,5,16].

Global and national public health bodies such as the World Health Organization and the Centers for Disease Control and Prevention have increasingly adopted verified social media accounts to strengthen their public reach and credibility [1,7]. These verified profiles help distinguish official sources from informal or potentially misleading accounts, reducing the risk that the public will rely on unauthorised or inaccurate information [1,7]. During health emergencies, these organisations have used their verified accounts to share updates on disease trends, recommended preventive measures, and policy changes, often in multiple languages and formats such as short videos, infographics, and thread‑style posts [1,7]. By centralising information through trusted accounts, agencies aim to create a consistent reference point that individuals can return to when seeking reliable guidance [1,7]. Verified accounts have also allowed these organisations to engage directly with the public, answering questions, clarifying ambiguities, and monitoring public sentiment about different health issues [1,7]. Work on health‑promotion frameworks has highlighted how such accounts can be used to build long‑term trust by providing clear, consistent, and evidence‑based messaging over time, rather than only during crises [3,7]. At the same time, these accounts must navigate the challenge of competing narratives and misinformation, which requires ongoing monitoring and rapid response when false claims appear [2,7]. Later analyses of social‑media‑based health communication have shown that verified accounts function best when they are part of a broader strategy that includes collaboration with local partners and adaptation to local contexts [1,2,7].

To support the growing role of social media, many health institutions have established dedicated digital or social media communication units responsible for planning, implementing, and monitoring online activities [16,17]. These units typically bring together communication specialists, data analysts, and sometimes behavioural scientists to coordinate messaging across platforms and align it with public health objectives [16,17]. They often develop content calendars, design visual materials, and respond to public comments, ensuring that communication is consistent, timely, and responsive to emerging issues [16,17]. In some cases, digital‑communication teams also work alongside epidemiologists and risk‑communicators to ensure that posts are scientifically accurate and sensitive to the emotional and cultural context of the audience [16,17]. The creation of such units reflects a recognition that managing social media is not simply a technical task but a specialised profession that requires ongoing training and coordination [17,18]. Reviews of ethics, transparency, and AI-based tools in healthcare communication have argued that clear governance structures are needed to address issues such as privacy, data use, and the potential for automated content to amplify bias or misinformation [17,18]. In practice, digital‑communication units have also been tasked with monitoring public sentiment during emergencies, identifying emerging concerns, and adjusting messaging to counter confusion or misinformation [17,18]. Taken together, the institutionalisation of these units shows that social media has moved from being a side project for a few enthusiastic staff to an integral part of organisational capacity for public health communication [16-18].

During global health emergencies such as the COVID‑19 pandemic, social media became a central channel for public health communication, with agencies using it to share updates, guidelines, and policy changes in near real time [4,19]. Platforms were also used to correct misinformation, clarify public confusion, and reassure communities during periods of uncertainty [4,19]. Systematic work on pandemic‑related communication has shown that social media allowed agencies to reach large and diverse audiences quickly, particularly when traditional media channels were slower or less accessible [5,19]. At the same time, the same platforms amplified conflicting narratives, rumours, and false claims about treatments, vaccines, and prevention measures, which required agencies to devote significant effort to monitoring and responding [4,19]. In response, many organisations intensified their use of verified accounts, coordinated messaging across platforms, and collaborated with local partners to ensure consistency in key messages [5,19]. Reviews of COVID‑19‑related public‑health responses have highlighted how digital‑communication units played a critical role in tracking public sentiment, identifying emerging misinformation, and adjusting strategies to maintain trust [5,19]. From an institutional perspective, the experience of the pandemic accelerated the formal integration of social media into emergency‑response frameworks, as agencies recognised that online communication was no longer optional but a core component of crisis management [4,5,19].

In the years following major health emergencies, public health agencies have increasingly relied on social media to foster public engagement, respond to questions, and demonstrate transparency [3,12]. Platforms allow agencies to explain the reasoning behind policy decisions, share data in more accessible formats, and invite feedback from the communities they serve [3,12]. This has helped shift the relationship between public health institutions and the public from one‑way information dissemination toward a more interactive dialogue, where users can ask questions, express concerns, and sometimes co‑shape future communication strategies [3,12]. At the same time, social media has become a tool through which the public can hold institutions to account, demanding clarification, consistency, and responsiveness when guidance appears unclear or contradictory [3,8].

Social media as a real‑time data source for disease surveillance and outbreak detection

User‑Generated Content as Epidemiological Data

In recent years, public health researchers have begun to treat social media posts as a new kind of epidemiological signal, alongside more traditional indicators such as clinical records and laboratory reports [4,20,21]. When people write about symptoms, diagnoses, or health‑related experiences online, they generate digital traces that can be mapped to time, location, and language, making these traces potentially useful for tracking disease activity [20,21]. User‑generated content often appears very close to the time when symptoms start, which means that platforms can capture what people are feeling and doing before they seek formal care or are captured in official datasets [20,21]. This temporal proximity has led some analysts to argue that social media can act as an early warning system, especially in settings where health-information systems are slow or fragmented [4,20]. At the same time, social media content is not a direct substitute for clinical confirmation; it must be interpreted alongside other data sources [4,20,21]. Studies of symptom reporting on platforms have shown that posts may reflect a mix of confirmed illness, self‑diagnosis, anxiety, and even misinterpretation, which introduces uncertainty into any analysis [20,21]. Nevertheless, when aggregated across large numbers of users and combined with statistical models, such content can reveal patterns, such as increases in mentions of cough, fever, or fatigue over time or in particular regions, that coincide with known outbreaks [4,20,21]. Reviews of outbreak‑surveillance experiences have highlighted that, while social media cannot replace formal testing, it can help identify areas where more intensive investigation may be needed, guiding the deployment of resources and alerting local health authorities to emerging clusters [4,20,21].

Hashtags and keywords have become key tools for transforming unstructured social media content into meaningful signals for disease surveillance [20-22]. Platforms such as Twitter and similar microblogging services often organise conversations around specific tags, allowing analysts to isolate health‑related discussions from broader online chatter [21,22]. By identifying recurring terms such as #flu, #COVID, or location‑specific combinations, researchers can estimate the volume and geographic spread of disease‑related conversations over time [21,22]. In some cases, these keyword‑based approaches have been shown to mirror or precede increases in official case counts, especially during outbreaks of respiratory or gastrointestinal diseases [20-22]. Beyond single words, more sophisticated keyword lists can capture variations in symptom descriptions, home remedies, and concerns about specific interventions, which helps refine the signals extracted from social media [20,22]. Work on mining social media for public health monitoring has demonstrated that carefully curated lexicons, often built from previous outbreaks or local clinical vocabulary, can improve the accuracy of outbreak detection and reduce noise from unrelated topics [20,22]. At the same time, these approaches must account for non‑medical uses of the same terms, such as metaphorical references to illness or unrelated commercial content, which can distort the apparent burden of disease [21,22]. Ongoing research has, therefore, focused on combining keyword‑based tracking with contextual filters, temporal smoothing, and integration with conventional surveillance data to turn simple hashtag counts into more robust indicators of public health relevance [20-22].

Crowdsourced health data drawn from social media can act as a supplement to traditional reporting systems, especially in settings where routine surveillance is slow, incomplete, or geographically uneven [20,23,24]. When individuals share information about illness, exposure, or care‑seeking behaviour online, they effectively contribute to a distributed form of disease monitoring that is not centrally organised but still strategically useful [23,24]. In some cases, this crowdsourced information has revealed hotspots of concern that were not yet evident in official statistics, prompting further investigation or targeted interventions [20,23,24]. However, relying on crowdsourced data also introduces important limitations [20,23,24]. Digital divides mean that certain populations, such as older adults, rural communities, or those with limited internet access, may be under-represented, which can skew the apparent distribution of disease [23,24]. At the same time, the informal nature of user‑generated content means that self‑reports are not always medically accurate, and some posts may conflate symptoms or exaggerate severity [20,23]. Reviews of social‑media‑based health reporting have, therefore, stressed that these data work best when treated as complementary signals that add timeliness and breadth rather than as standalone evidence [20,23,24]. When combined with traditional surveillance, such as case reports and laboratory confirmations, crowdsourced signals can help public health agencies identify emerging trends, verify local concerns, and allocate resources more efficiently [20,23,24].

One of the most discussed advantages of social media for disease surveillance is its timeliness compared with conventional reporting systems [21,23,25]. In many settings, clinical data follow a cascade of steps, consultation, testing, diagnosis, reporting, and aggregation, each of which can introduce delays that reduce the usefulness of information for early‑warning purposes [21,23,25]. Social media, by contrast, often captures people’s experiences and concerns close to the time they occur, sometimes even before formal diagnosis, creating a shorter feedback loop between illness and detection [21,25]. This time advantage has been especially relevant during rapidly evolving outbreaks, where even a few days’ lead can make a difference in containment and public‑health response [21,23,25]. Analyses of outbreak‑notification timelines have shown that social media signals can arise earlier than official case reports, particularly in regions with limited diagnostic capacity or weak reporting infrastructure [21,23,25]. By monitoring user‑generated content, analysts have been able to detect subtle increases in symptom‑related posts days before they appear in surveillance dashboards, suggesting that platforms can function as a leading indicator of disease activity [21,23,25]. However, this lead time also comes with the risk of false alarms, as spikes in online discussion can reflect anxiety, media coverage, or rumours rather than actual increases in disease transmission [23,25]. Reviews of digital surveillance systems have therefore emphasised the need to combine the timeliness of social media with rigorous validation steps, such as comparison with clinical data and temporal‑spatial consistency checks, to ensure that early signals are meaningful rather than misleading [21,23,25].

Several case studies have illustrated how early outbreak signals have been detected or reinforced through social media, even before formal systems flagged them [4,25,26]. In one example, spikes in location‑specific symptom‑related posts were observed in advance of an official confirmation of a respiratory outbreak, prompting local teams to intensify testing and surveillance in those areas [4,25]. Similar experiences have been reported in other contexts, where community‑level discussions about unusual clusters of illness or concern over specific facilities coincided with later‑confirmed events [25,26]. These examples suggest that social media can act as a “sensing” layer, drawing attention to patterns that might otherwise be overlooked because of reporting gaps or delayed data entry [4,25,26]. What these case studies also show is that social‑media‑based signals are most useful when they are embedded within broader surveillance ecosystems rather than treated in isolation [4,25,26]. Work describing outbreak-detection systems has highlighted how early social media alerts were often followed by field investigations, laboratory testing, and cross-checks with hospital records to confirm the presence of disease [4,25,26]. In some instances, integration with traditional systems allowed agencies to respond more quickly to emerging clusters, reducing the window between community concern and official action [4,25,26]. At the same time, some cases have also revealed the limitations of relying on informal reports, for example, when online discussions reflected fear or misinformation rather than true outbreaks, reinforcing the need for careful validation and contextual interpretation [4,25,26].

Integration With Traditional Disease Surveillance Systems

Digital epidemiological methods, including social media monitoring, are increasingly being positioned as a complement to traditional surveillance rather than a replacement [23,27]. Traditional systems rely on clinical data, laboratory reports, and structured surveys, which are relatively slow but standardised and reliable, while digital approaches can capture real‑time, community‑level discussions about symptoms and health concerns across large populations [23,27,28]. By combining both, public health teams can triangulate information: digital signals can highlight where to look, and traditional methods can confirm whether an actual change in disease burden is occurring [23,27,28]. This complementarity allows agencies to detect early anomalies, track public sentiment, and adjust communication strategies more quickly than they could using only conventional reporting [23,27,28]. In practice, this hybrid approach has been used in outbreak detection and forecasting, where social media content is treated as an early‑warning layer that triggers more detailed investigation using clinical and laboratory data [23,27,28]. For example, when digital monitoring shows unusual symptom‑related discussions in a given region, authorities may increase testing or conduct field surveys to verify the situation [23,27,28]. At the same time, integrating digital and traditional methods requires careful attention to biases in participation, unequal internet access, and ethical concerns about privacy and surveillance [23,28]. Overall, the complementarity between the two approaches reflects a move toward more pluralistic, multi‑layered surveillance systems that draw on multiple sources of information to produce more timely and robust public health responses [23,27,28].

Hybrid surveillance models that integrate clinical data with social media content have begun to offer a more structured way of bringing digital signals into formal public health practice [29,30]. These models typically use laboratory-confirmed cases, hospital admissions, and syndromic surveillance as the core of the system, while adding social media-derived indicators as an additional layer that can be monitored in real time [29-31]. By aligning time stamps and geographic units across both data streams, analysts can examine whether rises or falls in symptom‑related posts correspond with changes in official case counts and use any discrepancies to guide further investigation [29-31]. In some implementations, such hybrid models have been used to track infectious diseases, monitor public response to vaccination campaigns, or detect clusters of illness that are not yet reflected in clinical reports [29-31]. For instance, when social media shows a surge in symptom‑related posts in a specific area but clinical data remain low, agencies may investigate possible under‑reporting, conduct rapid field surveys, or intensify community outreach to clarify the situation [29,30]. Conversely, when clinical data show rising cases but social media shows little discussion, agencies may assess whether communities are under‑informed or using other platforms to communicate [29,31]. These kinds of checks help ensure that digital signals are interpreted alongside more reliable, structured evidence, rather than treated as standalone proof of disease activity [29-31].

Dashboards and real‑time monitoring tools have become central to the integration of social media data into traditional surveillance workflows [28,29]. These platforms gather feeds from multiple sources, such as clinical reports, laboratory data, and social media streams, and present them in a single, visual interface that can be updated continuously [26,28,29]. By aggregating and mapping symptom‑related content, case counts, and testing rates, dashboards allow analysts to see emerging patterns, track changes over time, and compare different regions at a glance [26,28,29]. This kind of real‑time visualisation supports faster decision‑making, especially during outbreaks, when timely information is critical [26,28,29]. In practice, these tools have been used to monitor infectious disease trends, evaluate public response to health messages, and identify geographic areas where digital indicators diverge from official statistics [26,28,29]. For example, when a dashboard shows a spike in symptom‑related posts alongside rising case counts in a given region, agencies can prioritise that area for targeted interventions, such as enhanced testing, community education, or resource allocation [28,29]. Dashboards can also help teams monitor misinformation or public concern, allowing them to adjust communication strategies in response to changing sentiment [26,28,29]. Overall, the use of dashboards and monitoring tools represents a move toward more operationalised integration of social media into routine surveillance, where digital signals are not just observed but actively incorporated into the day‑to‑day work of public health teams [26,28,29].

Despite the promise of social media as a surveillance tool, validating the data it produces remains a significant challenge [22,24]. Unlike clinical or laboratory data, which are standardised and collected under controlled conditions, social media content is generated voluntarily, often without clear context, and can be influenced by factors such as media coverage, jokes, or viral posts that are not directly related to actual disease activity [21,22,24]. This means that spikes in symptom‑related posts may reflect heightened public concern or misinformation rather than a real increase in illness, making it difficult to interpret signals without further verification [21,22,24]. Validation also depends on how well digital signals align with traditional data sources, but this is not always straightforward [21,22,24]. Participation on social media is uneven, favouring younger, urban, and more digitally connected populations, so the data may not be representative of the whole population or of high‑risk groups [21,22,24]. In addition, self‑reported symptoms can be inaccurate, exaggerated, or metaphorical, and some users may not use health‑related terms in the ways that analysts expect [21,22,24]. These issues mean that drawing firm conclusions from social media data alone can be misleading and that any signals extracted from these platforms must be treated as provisional until they are supported by more reliable evidence [21,22,24]. For these reasons, the literature on digital surveillance consistently emphasises the need for robust validation frameworks, transparent methods, and close integration with clinical and epidemiological data to ensure that social media-derived indicators are both meaningful and trustworthy [21,22,24].

Government and international public health agencies play a crucial role in determining how and whether integrated surveillance systems that combine social media with traditional data are adopted and scaled up [23,26,30]. These organisations have the authority to set standards, allocate resources, and coordinate cross‑jurisdictional efforts, which is essential for creating coherent, multi‑layered surveillance infrastructures that can operate across regions and countries [23,26,30]. In practice, the willingness of agencies to invest in digital surveillance tools, training, and governance frameworks often shapes how quickly and systematically these approaches are embedded into routine public health practice [23,26,30]. Several studies highlight examples in which national or global agencies have begun to pilot hybrid systems, sometimes in collaboration with academic partners, digital‑health firms, or civil‑society actors [23,26,30]. These efforts have focused on using dashboards, automated monitoring, and validation protocols to bring social media signals into alignment with clinical and epidemiological data, while also addressing privacy, ethical, and technical challenges [23,26,30]. At the same time, uptake has been uneven, as some institutions remain cautious about the reliability of digital data, the potential for misuse, or the resource requirements involved in building and maintaining such systems [23,26,30]. By setting clear guidelines, supporting capacity building, and fostering collaboration across sectors, governments and international bodies can help ensure that integrated surveillance remains evidence‑based, transparent, and oriented toward equitable public health outcomes [23,26,30].

Methodological approaches to using social media data for epidemiological surveillance

Data Mining and Natural Language Processing Techniques

Keyword filtering is one of the most basic but widely used methods for extracting health‑related content from large volumes of social media data [21,22]. When researchers are interested in tracking disease-related discussions, they begin by defining a list of terms such as symptom names, disease labels, or medication names and then scan posts for the presence of these keywords [21,22,32]. This approach allows analysts to quickly narrow down vast amounts of unstructured text to a more manageable subset that is likely to be relevant, acting as a first pass before more detailed analysis [21,22,32]. Keyword‑based extraction is especially useful in early warning systems, where the goal is to detect unusual spikes in mentions of specific health terms or phrases that may signal emerging concerns [21,22]. However, relying only on keywords introduces several limitations [21,22,32]. The same term can be used in multiple ways: to describe genuine illness, to make jokes, or to refer to unrelated topics, which can produce false signals if not checked against context [21,22,32]. In addition, disease‑related discussions may use synonyms, slang, or local expressions that are not captured by a predefined keyword list, leading to missed information [21,22,32]. Despite these constraints, keyword filtering remains a practical starting point in many studies, particularly when combined with more advanced methods such as machine learning or topic modeling that can refine the selection of relevant content and reduce noise [21,22,32].

Sentiment analysis is a method used to assess the emotional tone of social media content, helping researchers understand how the public perceives specific diseases, treatments, or public health interventions [21,32]. Researchers apply algorithms that classify text as positive, negative, or neutral based on patterns in word use and then aggregate these classifications across large sets of posts [21,32]. This allows them to see whether discussions around a particular disease are dominated by fear, anger, hope, or trust, which can be valuable for designing communication strategies [21,32,33]. When applied to health topics, sentiment analysis can reveal how public reactions shift over time, such as when new information emerges, policies change, or controversy arises around a vaccine or treatment [21,32,33]. For example, a sudden increase in negative sentiment after a public announcement may signal confusion or resistance, prompting agencies to adjust messaging or provide additional clarification [32,33]. At the same time, sentiment analysis is not a perfect mirror of public opinion, since online expression can be influenced by humour, sarcasm, and online communities that amplify extreme views [21,32,33]. This means that the results must be interpreted carefully and combined with other forms of data, such as survey responses or qualitative interviews, before drawing strong conclusions about population attitudes [32,33].

Topic modeling is a statistical technique that allows researchers to discover hidden thematic structures in large collections of social media posts without having to read each one manually [22,32,34]. By analysing patterns of word co‑occurrence, these methods group documents into clusters of related topics, each representing a broad theme such as vaccination, symptoms, side effects, or treatment experiences [22,32,34]. This approach is especially useful in public health because it can reveal emerging concerns that were not anticipated by the analyst, such as new side‑effect narratives, local myths, or regional patterns of fear and uncertainty [22,32,34]. In practice, topic‑modeling outputs are often used as a first step in exploratory analysis, helping researchers decide which areas deserve closer attention [22,32,34]. For example, when a cluster of posts repeatedly mentions headache and fatigue alongside a specific vaccine name, this may prompt further investigation into whether there is a structured narrative forming around those symptoms [22,32,34]. At the same time, topic‑modeling results are probabilistic and depend on the quality of the input data, including how posts are cleaned, filtered, and pre‑processed [22,32,34]. The models are also sensitive to language variation, so they may need to be adapted for different languages or cultural contexts to avoid misinterpreting the underlying themes [22,32,34]. Despite these limitations, topic modeling has become a key tool for moving beyond simple keyword counts to a more nuanced understanding of what health topics are circulating in online spaces [22,32,34].

Machine learning models have become central to the task of automatically classifying which social media posts are relevant to health and which can be treated as noise [22,29,35]. Researchers train classifiers on labelled datasets where human annotators have marked posts as disease‑related, non‑health, or irrelevant, allowing algorithms to learn patterns that distinguish genuine health content from other types of discussion [22,29,35]. Once trained, these models can process large volumes of text far more quickly than human coders, making them indispensable for real‑time surveillance and monitoring applications [22,29,35]. Different types of models can be used depending on the research question and data conditions, including rule‑based classifiers, supervised learning algorithms, and deep‑learning approaches [22,29,35]. Supervised models, for example, can be optimised to recognise context‑specific terms, negations, and metaphors that change the meaning of health‑related expressions, reducing false positives and false negatives [22,29,35]. At the same time, these models are not infallible; they depend heavily on the quality and representativeness of the training data, and they may perform poorly when applied to new populations, languages, or emerging topics that were not present in the original dataset [22,29,35]. For this reason, current work in digital epidemiology emphasises the need for continuous retraining, validation, and close collaboration between data scientists and public health experts to ensure that classification models remain accurate and meaningful [22,29,35].

Large social media datasets are inevitably full of noise, including irrelevant content, jokes, sarcasm, and unrelated discussions that can distort the apparent pattern of health‑related activity [21,22,24]. Handling this noise is a major challenge in any attempt to use social media for surveillance, because noise‑laden signals can lead to false alarms or missed signals if not managed carefully [21,22,24]. Researchers therefore combine multiple techniques, such as keyword filtering, machine learning classification, and manual review, to remove or down‑weight posts that are unlikely to reflect genuine health concerns [21,22,24]. Noise‑reduction strategies also depend on how the data are structured and what the research question demands [21,22,24]. For example, removing posts that contain only generic or commercial content, or that are written in languages that are not under study, can help focus the analysis on meaningful exchanges [21,22,24]. In some cases, analysts introduce confidence thresholds so that only posts classified as health‑related with high probability are included in the final analysis, further reducing the risk of misinterpreting random noise as a signal [21,22,24]. At the same time, over‑filtering can remove genuine but unusual expressions, so the choice of thresholds must strike a balance between sensitivity and specificity [21,22,24]. Overall, the literature on social media‑based surveillance treats noise handling not as a one‑time step, but as an ongoing process that must be transparent, documented, and integrated into the broader methodological design [21,22,24].

Geospatial and Predictive Analytics in Digital Epidemiology

Geotagged social media data have become an important source of spatial information for mapping how diseases may be spreading across regions [23,36]. Posts that include location tags allow researchers to assign symptom‑related or disease‑related content to specific geographic areas, such as cities, districts, or even smaller neighbourhoods [23,34,36]. This spatial precision enables analysts to visualise where people are reporting unusual symptoms or health concerns and how these patterns change over time [23,36]. In practice, geotagged posts have been used to create maps that highlight clusters of illness‑related discussions, which can help public health teams identify potential hotspots before these areas appear clearly in traditional surveillance systems [23,34,36]. Because geotagged data provide both time and location information, they support spatiotemporal analysis, allowing researchers to trace the movement of health‑related conversations over days or weeks [23,34,36]. In some cases, these patterns have aligned with later increases in confirmed cases, suggesting that location‑tagged social media content can function as an early‑warning layer for disease spread [23,34,36]. However, the usefulness of geotagged data is limited by how many users actually share location information, which varies across platforms, cultures, and age groups [23,34,36]. Despite this, when combined with other geographic data sources, geotagged social media can help refine the spatial resolution of surveillance systems and improve the targeting of public health interventions [23,34,36].

Spatial clustering techniques have become central to detecting potential outbreaks from social media data by identifying where health‑related posts are concentrated more than expected by chance [23,27,28]. These methods examine how symptom‑related or disease‑related content is distributed across space and look for “hotspots” where the density of posts is unusually high, which may indicate emerging disease activity or increased public concern [23,27,28]. By applying algorithms that measure spatial dependence and cluster significance, researchers can distinguish random variation from patterns that warrant closer inspection [23,27,28]. In practice, these spatial clustering approaches have been used alongside traditional epidemiological data to detect where outbreaks might be starting or intensifying [23,27,28]. For example, when social media posts mentioning respiratory symptoms show strong clustering in a particular region, public health teams can prioritise that area for enhanced testing, field investigations, or community outreach [23,27,28]. At the same time, the performance of these techniques depends on the quality and representativeness of the underlying data, since uneven participation and sampling biases can distort the apparent pattern of clusters [23,27,28]. When used carefully, spatial clustering methods help turn noisy social media content into structured spatial signals that can be integrated into broader surveillance thinking and decision‑making [23,27,28].

Time‑series models that incorporate social media data have been used to forecast disease trends by tracking how symptom‑related or disease‑related discussions change over successive days or weeks [21,28,30]. Researchers have applied statistical and machine‑learning methods to sequences of social‑media counts, such as the number of posts mentioning specific symptoms or conditions, and then used these series to predict future case levels or health‑related behaviours [21,28,30]. In many cases, changes in social media activity have preceded or closely mirrored official case reports, suggesting that these models can capture early shifts in public health dynamics [21,28,30]. One advantage of time-series approaches is that they can be calibrated to historical data, allowing analysts to assess how well past social media patterns predicted later outcomes and then improve their models accordingly [21,28,30]. For example, during infectious disease events, models have used social‑media volumes alongside clinical data to forecast short‑term case trajectories, which can inform decisions about resource allocation, staffing, and communication strategies [21,28,30]. However, these predictions are only as good as the underlying assumptions and the stability of the relationship between social media behaviour and actual disease activity [21,28,30]. When external factors such as media coverage, policy announcements, or online campaigns influence what people post, the relationship can break down, requiring frequent recalibration and careful interpretation of results [21,28,30].

Artificial intelligence (AI) techniques have increasingly been integrated into disease‑forecasting systems that draw on social media to improve the accuracy and responsiveness of predictions [29-31]. Machine‑learning models trained on large volumes of social‑media text, temporal patterns, and sometimes location data can identify complex, non‑linear relationships between online activity and future case trends, outperforming simpler statistical approaches in some settings [29-31]. These AI‑driven systems can automatically update their forecasts as new data arrive, which is particularly useful during rapidly evolving outbreaks when decision‑makers need timely information [29-31]. In practice, AI‑based models have been used to forecast infectious disease incidence, anticipate spikes in health‑seeking behaviour, and estimate the impact of public‑health interventions, often using combinations of social‑media signals, clinical data, and contextual variables such as mobility patterns or weather conditions [29-31]. By learning from past events, these systems can generalise across different regions and settings, although their performance depends heavily on the quality and representativeness of the training data [29-31]. Public health teams can then use these forecasts to guide preparedness activities, such as increasing testing capacity, adjusting communication strategies, or targeting specific communities [29-31]. Nevertheless, the opacity of some AI models and the risk of amplifying biases in social media participation mean that these tools must be transparently validated and used as part of broader, human‑led decision‑making processes rather than as standalone or opaque oracles [29-31].

Despite the promise of geospatial analysis, the accuracy and coverage of location data in social media present important limitations for epidemiological surveillance [21,23,24]. Only a subset of users share geotagged posts, and many platforms rely on user‑declared locations or coarse‑grained information such as city or country, which can misrepresent where people actually are [21,23,24]. This unevenness means that spatial patterns derived from social media may reflect where people choose to share their location rather than where disease activity is truly occurring [21,23,24]. As a result, maps and clusters based on location‑tagged content can be biased toward certain age groups, socioeconomic levels, or urban areas while under‑representing rural, older, or less‑connected populations [21,23,24]. Coverage problems are further compounded by the fact that participation on social media varies across regions and cultures, leading to uneven geographic representation in the data [21,23,24]. In some countries or communities, low internet penetration or limited use of particular platforms can mean that location‑based surveillance captures only a small fraction of the population, reducing the reliability of any spatial inferences [21,23,24]. At the same time, privacy‑related settings and platform‑level changes can suddenly alter how much location information is available, making it difficult to ensure consistent monitoring over time [21,23,24]. For these reasons, studies emphasise that location‑based surveillance should be treated as a partial, complementary layer of information rather than a complete picture of disease geography and that any spatial findings must be interpreted alongside more representative data sources whenever possible [21,23,24].

Effectiveness of social media in monitoring and predicting infectious disease trends

Empirical Evidence From Case Studies (Influenza, COVID‑19)

Several empirical studies have sought to compare how closely social media-derived indicators align with official epidemiological data for infectious diseases, particularly influenza and COVID‑19 [21,37,38]. These comparisons typically involve mapping time‑series patterns of symptom‑related posts, search queries, or hashtags onto laboratory‑confirmed case counts, hospitalisations, or surveillance metrics from national or subnational health agencies [21,37,38]. In many cases, researchers have found moderate to strong correlations between digital signals and conventional statistics, suggesting that social media can capture aspects of disease activity even when it does not capture every case [21,37,38]. However, the strength of this alignment tends to vary by context, disease, and platform [21,37,38]. For example, during some influenza seasons, social media spikes in symptom‑related content occurred shortly before or alongside official increases in outpatient visits or laboratory reports, whereas in other settings the correspondence was weaker or more delayed [21,37,38]. Similarly, during parts of the COVID‑19 pandemic, certain countries showed clearer links between online discussions and confirmed case trajectories than others, likely due to differences in testing capacity, social media use, and public‑health communication practices [21,37,38]. Overall, these comparative studies suggest that social media data are neither a perfect mirror nor a reliable standalone indicator, but rather a supplementary source that can enrich the understanding of infectious disease trends when used alongside and validated against traditional surveillance outputs [21,37,38].

Twitter has been one of the most widely studied platforms for predicting influenza trends, because of its public, real‑time feed and the relative ease of collecting and analysing its content [21,37,39]. Researchers have used tweets mentioning symptoms such as cough, fever, or specific illness terms, along with hashtags like “flu” or "influenza", to track how public discussion about the disease rises and falls over time [21,37,39]. By applying natural language processing and machine‑learning techniques, analysts have been able to filter out irrelevant posts and classify the remaining content according to likely health relevance, generating time‑series indicators that can be compared with official influenza surveillance data [21,37,39]. In practice, several studies have shown that Twitter‑based indices can capture seasonal influenza patterns with reasonable accuracy, sometimes detecting peaks slightly earlier than traditional reporting systems [21,37,39]. This timeliness advantage has made Twitter attractive as a real‑time layer of information that can complement laboratory‑confirmed case counts and sentinel‑surveillance networks [21,37,39]. For example, when social media activity about influenza‑like symptoms rises in a given region, public health teams can interpret this as a signal that local disease activity may be increasing and respond by reinforcing surveillance, increasing testing, or adjusting vaccination campaigns [21,37,39]. At the same time, these models are not error‑free, and the correspondence between Twitter‑derived signals and clinical data can weaken in settings with low Twitter penetration, high levels of noise, or campaigns that alter how people talk about the disease online [21,37,39]. Despite these limitations, the evidence suggests that Twitter‑based trend prediction has become a useful, though provisional, component of modern influenza surveillance frameworks [21,37,39].

During the COVID‑19 pandemic, digital signals from social media and related platforms were increasingly used to track case trends and public responses in real time [25,37,40]. Researchers and public health teams have analysed posts mentioning symptoms, test results, quarantine measures, and vaccination experiences to map where concern and illness‑related discussion were concentrated [25,37,40]. In many cases, spikes in such content have coincided with or preceded official reports of rising case numbers, suggesting that online discussions can function as an early layer of situational awareness even when formal reporting is lagging or incomplete [25,37,40]. These digital signals have also been used to understand how public sentiment and behaviour changed during different phases of the pandemic, including lockdowns, vaccination rollout, and the emergence of new variants [25,37,40]. For example, increases in posts expressing anxiety, frustration, or uncertainty often aligned with periods of policy change or rising case counts, indicating that online content can reflect the psychological and social dimensions of the outbreak as well as its clinical trajectory [25,37,40]. In some settings, analysts have combined social‑media‑derived indicators with mobility data, search queries, and clinical records to build more holistic models of transmission dynamics and public response patterns [25,37,40]. However, interpreting these signals requires care, because not all posts describe genuine illness, and factors such as media coverage or misinformation campaigns can artificially inflate discussion without corresponding changes in actual disease burden [25,37,40]. Overall, the experience with COVID‑19 shows that digital signals can support more responsive, context‑rich surveillance, but they must be used alongside, and validated against, more structured epidemiological data [25,37,40].

The empirical record of social‑media‑based infectious disease prediction includes both notable successes and clear failures, highlighting the strengths and limitations of these approaches [21,41,42]. Some studies have demonstrated that carefully designed models can forecast influenza or COVID‑19 trends with reasonable accuracy by combining symptom‑related posts, search data, and machine‑learning algorithms, often outperforming simpler benchmarks or even capturing peaks slightly earlier than official reports [21,41,42]. In these cases, the models were usually grounded in high‑quality training data, clear definitions of relevant terms, and close alignment between digital and traditional surveillance periods [21,41,42]. However, other experiences have shown that the same methods can fail when applied to different contexts, diseases, or time windows [21,41,42]. Models that worked well during one influenza season have sometimes underperformed in the next, and indices that correlated strongly with COVID‑19 cases in one country have shown weaker or inconsistent relationships elsewhere [21,41,42]. These inconsistencies arise because social media behaviour is influenced by many factors beyond disease activity, including public-health messaging, media coverage, and platform-specific dynamics [21,41,42]. In some cases, algorithms misclassifying posts or misinterpreting context have led to over‑ or under‑estimates of disease burden, reducing the usefulness of the predictions [21,41,42]. The lesson from these mixed results is that prediction models built on social media must be treated as context-dependent hypotheses rather than universal tools and that their outputs should be continuously validated against clinical data and adjusted as new information becomes available [21,41,42].

Cross‑country comparisons of social‑media‑based surveillance have revealed important differences in how effectively digital signals can monitor infectious disease trends [37,39,40]. In high-income countries with strong internet penetration and established surveillance systems, social media often performs well as a supplementary or early-warning layer because platform use is widespread and relatively stable [37,39,40]. In some of these settings, correlations between symptom‑related posts and official case counts have been strong enough to justify integrating digital indicators into routine monitoring frameworks [37,39,40]. In contrast, in low- and middle-income countries, the same methods have sometimes produced weaker or more variable results due to differences in internet access, social media use, language diversity, and the structure of health-information systems [37,39,40]. For example, where only a small proportion of the population uses certain platforms or where multiple platforms coexist with different user profiles, social‑media‑derived signals may not reflect the broader population and can introduce bias into estimates [37,39,40]. Additionally, differences in how people talk about illness, how governments communicate risk, and how traditional surveillance is organised can all shape how closely online discussions match official disease patterns [37,39,40]. These cross‑country variations show that effectiveness is highly context‑dependent and that models developed in one country or setting cannot be automatically transferred to another without careful local adaptation and validation [37,39,40].

Accuracy, reliability, and validity of social media-based predictions

Social media-based predictions are often undermined by false positives and the spread of misinformation, which can distort the apparent pattern of disease activity [21,22,43]. Posts that exaggerate symptoms, mimic illness narratives for humour, or echo viral rumours can create artificial spikes in symptom‑related content that do not correspond to real increases in disease burden [21,22,43]. When these posts are treated as raw signals, the resulting models may overestimate the scale or timing of outbreaks, leading to misinformed decisions about resource allocation or public communication [21,22,43]. In some cases, misinformation campaigns around vaccines, treatments, or prevention measures have also altered how people talk about disease, further complicating the interpretation of online discussions [22,43]. At the same time, the way algorithms and platform features shape content visibility can amplify certain types of posts while marginalising others, creating a biased picture of what is happening in the real world [21,22,43]. Automated detection systems may struggle to distinguish between genuine health reports and irony, exaggeration, or speculative content, especially when they rely heavily on keywords rather than deeper contextual analysis [21,43]. This means that the accuracy of social media-based predictions often depends as much on the quality of the filtering and interpretation methods as on the volume of data itself [21,22,43]. As a result, many researchers argue that social media signals should be treated as a noisy, imperfect layer that requires careful cleaning, validation, and cross‑checking with more reliable sources rather than as a direct measure of disease activity [21,22,43].

Another major limitation of social media-based predictions is the bias introduced by uneven digital participation and demographics [21,24,39]. Platforms tend to be used more heavily by younger, urban, and more educated populations, while older adults, rural communities, and people with limited internet access are under‑represented or absent from the data [21,24,39]. When models rely solely on social media, they effectively ignore or underweight the health experiences of those groups, leading to estimates that may not reflect the true distribution of disease across the population [21,24,39]. In practice, this bias can mean that social media shows strong signals in certain regions or age groups but weak or no signals in others, even when disease activity is similar or even higher in the under‑represented areas [21,24,39]. For example, a spike in symptom‑related posts in a city may be highly visible, while a more severe outbreak in a rural area with low internet penetration may go largely unnoticed in the digital record [21,24,39]. This kind of bias not only reduces the reliability of predictions but can also reinforce existing health inequalities by directing attention and resources toward digitally visible populations while neglecting those who are less connected [21,24,39]. To address this, some studies have called for mixed‑method approaches that combine social media with more traditional data sources, such as community surveys or clinic‑based reporting, to ensure that models are as representative and equitable as possible [21,24,39].

Because social media‑based data are inherently noisy and incomplete, validation against clinical data has become a central part of assessing their reliability [29,38,44]. Researchers have used laboratory‑confirmed case counts, hospital admissions, and other forms of structured health‑information systems to test whether digital signals correspond with actual disease trends over time [29,38,44]. Correlation checks, time‑lag analyses, and cross‑validation procedures have been used to determine how closely symptom‑related posts or search patterns align with observed increases or decreases in cases, allowing analysts to calibrate their models and identify periods when the digital record is most informative [29,38,44]. In some studies, this kind of validation has revealed that social media-derived indicators perform well for certain diseases or periods but poorly for others, depending on how people actually use the platforms and how the health system is organised [29,38,44]. For example, indicators may track influenza or respiratory illness accurately in settings where online discussion reflects real‑world consultations, but they may underperform in contexts where people rarely post about health or where clinical data are delayed or incomplete [29,38,44]. Validation has also helped uncover biases and artefacts, such as spikes driven by media coverage rather than actual disease, showing that the relationship between online content and disease activity is context‑dependent rather than universal [29,38,44]. Overall, the use of clinical data as a benchmark underscores that social media-based predictions are not self-validating tools but hypotheses that must be continuously tested and refined against more reliable sources of evidence [29,38,44].

The statistical reliability of social media-based predictive models has been a major focus of recent work, as researchers try to determine whether the patterns they detect are stable and reproducible rather than random noise [41,42,45]. Many studies have used standard performance metrics such as correlation coefficients, mean absolute error, and root‑mean‑square error to assess how closely model outputs match observed disease trends and to compare different modelling approaches [41,42,45]. When these metrics are strong and consistent across multiple time windows and settings, they suggest that the underlying relationships between digital signals and disease activity are robust enough to support meaningful predictions [41,42,45]. However, other studies have shown that the same models can perform very differently in different contexts, raising questions about their generalisability [41,42,45]. For example, an algorithm that fits influenza data well in one country or season may fail to capture the same patterns in another, often because of changes in how people use social media, how they talk about illness, or how the health system reports cases [41,42,45]. In some cases, over‑fitting to training data has led to high performance in historical periods but poor accuracy when applied to new outbreaks, highlighting the need for rigorous out‑of‑sample testing and regular model updating [41,42,45]. These findings suggest that statistical reliability should be assessed not only in terms of pointwise accuracy but also in terms of how well models transfer across settings and time and how transparent and reproducible their methods are [41,42,45].

A central debate in the literature concerns whether social media-based methods should replace or simply complement traditional surveillance systems [37,44,46]. Some authors argue that the speed and breadth of digital data make them uniquely suited to early warning and real-time situational awareness and that they could eventually take on a larger share of the surveillance workload, especially in well-connected settings [37,44]. Others caution that the limitations of online data, such as bias, noise, and incomplete coverage, mean that they are best treated as an additional layer rather than a substitute for clinical and laboratory‑based reporting [24,44,46]. This second view sees social media as a tool that can highlight where to look, guide resource allocation, and refine communication strategies, but not as a stand‑alone basis for policy decisions [37,44,46]. In practice, many recent studies support a mixed‑method approach, in which social media‑derived signals trigger more detailed investigation using traditional methods and where the strengths of each system are leveraged in combination [37,44,46]. For example, an unusual spike in symptom‑related posts might prompt increased testing, field surveys, or intensive data collection in a given area, allowing agencies to confirm whether a real outbreak is occurring and to characterise its scale and severity [24,37,44]. This kind of workflow treats digital and traditional data as complementary, with each filling gaps in the other’s coverage and timing [37,44,46]. The ongoing debate therefore reflects a broader tension between the promise of rapid, scalable digital tools and the need for accurate, standardised, and equitable surveillance that can be trusted by policymakers, health professionals, and the public [37,44,46].

Social media as a tool for promoting health behaviour change and public health interventions

Digital Campaigns for Health Promotion and Disease Prevention

Social media has become a key channel for vaccination campaigns, allowing public health agencies and advocacy groups to reach large, diverse audiences quickly and at relatively low cost [2,47]. These platforms make it possible to share information about vaccine safety, effectiveness, and schedules in formats that are accessible and shareable, such as short posts, infographics, and short videos [2,47]. In many settings, national immunisation programmes have used social media to promote catch‑up campaigns, address hesitancy, and counter misinformation about particular vaccines [2,47,48]. Campaigns have also used social media to create interactive spaces where users can ask questions, receive tailored responses, and observe how others talk about their experiences with vaccination [47,48]. Engagement with these campaigns often increases when messages are framed in relatable, localised language and when trusted voices, such as health professionals or community leaders, share endorsements [2,47,48]. At the same time, the spread of vaccine‑related misinformation on the same platforms has made it necessary for these campaigns to be proactive, transparent, and responsive, addressing concerns as they emerge rather than waiting for rumours to solidify [2,48]. Overall, social media has shifted vaccination promotion from one‑way information‑giving toward a more dynamic, two‑way communication process that can both educate and listen to public concerns [2,47,48].

Social media has also been widely used to promote basic hygiene and other preventive behaviours, especially during infectious disease outbreaks such as COVID‑19 or seasonal respiratory illnesses [48-50]. Platforms have carried messages about handwashing, mask-wearing, distancing, and other actions that can reduce transmission, often using simple, action‑oriented language and clear visuals [48-50]. In some cases, campaigns have combined these messages with reminders about symptoms and guidance on when to seek care, reinforcing the idea that individual behaviour is linked to broader community safety [48-50]. The interactivity of social media allows these messages to be adapted to different audiences and to be shaped by public feedback [48,49]. For example, when users express confusion about particular measures or report barriers to following them, subsequent posts can clarify or offer practical tips, such as ways to wash hands in settings with limited water or how to fit masks comfortably [48-50]. At the same time, digital campaigns have also had to contend with competing narratives, including jokes, scepticism, or outright rejection of preventive measures, which can dilute the impact of official messages [48-50]. Public health actors have, therefore, aimed to make hygiene-promotion content engaging, relatable, and culturally sensitive, using formats that encourage sharing and repetition within networks [48-50].

Visual content and storytelling have played a central role in making health‑promotion campaigns on social media more engaging and memorable [48,51]. Videos, infographics, and illustrated posts allow users to grasp complex information quickly and are often more likely to be shared than text‑heavy messages [48,51]. In many cases, campaigns have used short video narratives, testimonials, and dramatised scenarios to show how preventive behaviours can be incorporated into everyday life, making abstract health advice feel more concrete and achievable [48,49,51]. These narrative approaches also help humanise health messages by featuring real people, families, or health workers describing their experiences and motivations for adopting certain behaviours [48,49,51]. Such stories can reduce stigma, normalise help‑seeking, and encourage others to follow similar practices by showing that “people like them” are already doing so [48,49,51]. In addition, visual content can be adapted for different platforms, audiences, and languages, allowing campaigns to maintain a consistent core message while tailoring style and tone to local contexts [48,49,51]. Evidence suggests that posts combining clear visuals with simple, story‑based messaging often achieve higher reach and engagement, which can amplify the spread of preventive information within social networks [48,49,51].

Digital campaigns have increasingly focused on delivering targeted messaging to specific population groups, recognising that different audiences may respond to different framings and channels [47,50,51]. Social media platforms allow agencies to segment audiences by age, location, interests, or language, enabling them to design content that is more relevant to particular communities [47,50,51]. For example, vaccination or hygiene campaigns aimed at young adults often use informal language, memes, or popular music references, while those aimed at parents may emphasise child safety and family well-being [47,50,51]. In some settings, campaigns have also been tailored to reach vulnerable or underserved populations, such as older adults, rural communities, or minority groups, by using local languages, culturally familiar imagery, and trusted community figures as messengers [47,50,51]. These targeted approaches can help address specific concerns, such as mistrust of institutions, limited access to services, or competing priorities in daily life [47,50]. Evidence suggests that when messages are tailored to the lived realities and values of a particular group, they are more likely to be noticed, understood, and acted upon [47,50,51]. However, targeting must be done carefully to avoid stigmatising any group or reinforcing harmful stereotypes, and it should be guided by consultation with the communities it seeks to reach [47,50].

The effectiveness of social‑media‑based health‑promotion campaigns is often evaluated using engagement metrics such as likes, shares, comments, and views alongside more traditional indicators of behavioural change [47,48,52]. High engagement suggests that content has reached and resonated with users, but it does not automatically mean that people have changed their behaviours or beliefs [47,48,52]. In practice, studies have used these metrics to compare different types of posts, messages, or visual formats, identifying which combinations generate the greatest reach and interaction [47,48,52]. However, recent work has also highlighted the limitations of relying solely on engagement metrics, because they may reflect popularity more than impact [47,48,52]. A post may go viral because it is entertaining or controversial, even if it does not convey accurate health information or lead to improved outcomes [47,48,52]. To address this, some evaluations have combined digital engagement data with survey data, clinic records, or other sources to assess whether increased online activity corresponds with changes in knowledge, attitudes, or health practices [47,48,52]. Such mixed‑method approaches allow campaigns to distinguish between what is simply “liked” and what genuinely contributes to public health goals [47,48,52].

Behavioural Theories Applied in Social Media Interventions

The Health Belief Model has been frequently used as a framework for designing social media campaigns aimed at encouraging preventive health behaviours [2,49,50]. Under this model, people are more likely to take action if they believe they are at risk, see the seriousness of the problem, expect a benefit from acting, and feel that the barriers to action are manageable [2,49,50]. In digital campaigns, these concepts are often translated into messaging that highlights personal risk, consequences of inaction, and achievable steps that can be taken, such as vaccination, screening, or lifestyle changes [2,49,50]. By using relatable stories and visual content, practitioners try to increase perceived susceptibility and severity while offering practical solutions that reduce perceived barriers [2,49,50]. Researchers applying the Health Belief Model to social media interventions have found that structuring posts around perceived threat, perceived benefits, and cues to action can improve engagement and self‑reported intentions to change behaviour [2,49,50]. For example, campaigns that combine statistics about disease risk with personal narratives, clear advice, and simple actions (like booking a test or joining a vaccination programme) have been associated with higher levels of attention and willingness to act than more generic messages [2,49,50]. At the same time, these studies also show that the model cannot stand alone; it must be tailored to how people actually use social media, what they trust, and how they interpret risk in everyday life [2,49,50]. When used thoughtfully, the Health Belief Model offers a coherent way to design digital public‑health communication, but it must be adapted to the interactive, fast‑moving environment of social media rather than simply ported from traditional health‑education materials [2,49,50].

The Theory of Planned Behavior has been used to explain and shape health‑related behaviours by focusing on attitudes, perceived norms, and perceived behavioural control [47,48,52]. According to this theory, people are more likely to adopt a behaviour if they hold a positive attitude toward it, believe that important others approve of it, and feel that they have the ability and resources to carry it out [47,48,52]. In social media interventions, these elements are often embedded in posts that show positive outcomes, highlight community approval, and emphasise that the desired behaviour is feasible and supported [47,48,52]. For instance, messages about vaccination may stress how common it is for others to be vaccinated, frame it as a responsible choice, and provide practical steps for appointment booking, all of which can strengthen intention to act [47,48,52]. Studies applying this theory to digital campaigns have examined how online content influences perceived attitudes, social norms, and perceived control over actions such as physical activity, dietary change, or screening uptake [47,48,52]. In some cases, well‑designed social media messages that systematically address these three components have been linked to higher self‑reported intentions and, in a few trials, modest changes in behaviour over time [47,48,52]. However, the theory also highlights limitations: if people feel that structural barriers such as cost, access, or time are too high, simply changing attitudes or perceived norms may not be enough to produce sustained change [47,48,52]. For this reason, effective social media strategies guided by the Theory of Planned Behavior often combine persuasive messaging with links to concrete services, reminders, and support that help close the gap between intention and action [47,48,52].

Social cognitive theory has been used to understand how people learn health behaviours through observation, imitation, and feedback, principles that translate naturally to social media environments [2,48,51]. Observational learning can occur when users see others posting about their experiences with screening, vaccination, exercise, or healthy eating, and then consider whether to adopt similar actions [2,48,51]. In digital interventions, this is often operationalised by sharing stories, videos, or testimonials that show individuals successfully engaging in the target behaviour, overcoming obstacles, and experiencing positive outcomes, thereby modelling how such behaviour can be integrated into everyday life [2,48,51]. Research applying social cognitive theory to online platforms has shown that exposure to these kinds of models can increase self‑efficacy, motivate action, and reduce the sense that behaviour change is out of reach [2,48,51]. For example, campaigns that feature peers or trusted community figures describing how they started exercising, quit smoking, or managed a chronic condition have sometimes led to greater confidence among viewers that they too could make similar changes [2,48,51]. At the same time, the same principle can work in the opposite direction if users are exposed to pessimistic or fatalistic narratives that suggest health‑related action is futile or irrelevant [2,48,51]. This duality underlines the importance of designing digital content so that the models presented are relatable, credible, and aligned with public‑health goals rather than simply amplifying whatever stories happen to circulate online [2,48,51]. By harnessing observational learning deliberately, social media interventions can help people visualise pathways to change and see that healthy behaviours are achievable within their own social and cultural contexts [2,48,51].

Nudging and persuasive communication have become important tools in shaping health behaviours through social media [47,49,52]. Nudging, in this context, refers to small changes in how information is presented that steer people toward healthier choices without restricting their freedom, such as using default options, reminders, or salient visual cues [47,49,52]. On social media, nudges can appear in the form of prompts to book a vaccination appointment, follow‑up messages after a health post, or highly visible buttons that link directly to services, all of which reduce the effort required to act [47,49,52]. Persuasive communication complements nudging by crafting messages that appeal to emotions, social norms, or practical benefits in ways that resonate with specific audiences [47,49,52]. For example, posts that highlight the protection of family members, connect with cultural values, or break down complex health information into simple, actionable steps can increase the likelihood that people will engage with the content and take recommended actions [47,49,52]. Studies using these approaches have found that combining nudges with well‑designed persuasive messages can modestly improve behaviours such as appointment attendance, medication adherence, or engagement with preventive programmes, especially when messages are repeated and personalised [47,49,52]. However, both nudging and persuasion raise ethical considerations, as they can influence behaviour without always making the underlying mechanisms transparent, underscoring the need for transparency, consent, and ongoing evaluation in digital public health interventions [47,49,52].

Measuring behavioural outcomes in social media‑based interventions requires careful design and appropriate metrics, because engagement on a platform is not the same as actual health behaviour [50-52]. Many studies have relied on self-reported measures, such as survey responses about changes in dietary habits, physical activity, or healthcare-seeking behaviour, often collected before and after exposure to digital campaigns [50-52]. These self-reports can indicate shifts in attitudes or intentions, but they may overestimate real-world changes and are subject to social-desirability bias [50-52]. Researchers have therefore increasingly sought objective indicators, such as administrative records of vaccination uptake, screening attendance, or laboratory test ordering, to link online exposure to concrete health actions [50-52]. In some trials, digital campaigns have been connected to clinic‑level or system‑level data to assess whether higher engagement with social media content was associated with increased use of preventive services or changes in health‑related behaviour over time [50-52]. These mixed-methods approaches help bridge the gap between online activity and offline outcomes, though they remain challenging to implement consistently across different settings [50-52]. Additionally, short‑term follow‑up often limits the ability to detect sustained behaviour change, and there is still relatively little evidence on how long‑term or cost‑effective digital behavioural interventions really are [50-52]. Overall, the literature suggests that measuring behavioural outcomes in digital interventions is possible but requires rigorous methods, clear linkage between online content and real‑world data, and realistic expectations about the size and durability of effects [50-52].

The role of social media influencers and digital communities in shaping health behaviours

Influence of Social Media Personalities on Public Health Decisions

Social media influencers have increasingly shaped public attitudes toward vaccination, both positively and negatively [50,53,54]. Many followers view influencers as trusted, relatable figures whose opinions feel more personal than those of distant institutions, which can make vaccine‑related endorsements or critiques especially persuasive [50,53,54]. When influencers share personal stories of being vaccinated, explain how it fits into their daily lives, or highlight community benefits, studies have found that such narratives can strengthen intention to vaccinate and reduce hesitancy among their audiences [50,53,54]. In some campaigns, pairing influencers with public health messages has helped reach younger or otherwise hard‑to‑reach groups who rarely engage with official communication [50,53,54]. At the same time, when influencers express doubts about vaccines or promote unproven alternatives, those messages can amplify fear, erode trust in health authorities, and contribute to uneven vaccination coverage [51,54,55]. Because influencer content often spreads quickly and emotionally, brief, emotionally charged posts can have a disproportionate impact compared with longer, more technical public‑health materials [50,53,54]. Research on this dynamic suggests that the relationship between influencers and vaccination attitudes is powerful but inherently double‑edged: it can be harnessed for public‑health gain or exploited to deepen polarisation, depending on the credibility, motives, and messaging of the individuals involved [50,53,54].

Trust is a central factor in why social media influencers can shape health decisions, and how that trust is built and maintained has important implications for public health [54-56]. Many followers perceive influencers as more authentic and accessible than traditional health experts, especially when they share personal experiences, vulnerabilities, and everyday routines rather than formal, clinical information [54-56]. This sense of intimacy can make followers more likely to adopt health‑related recommendations, even when the influencer is not a medical professional [54-56]. Over time, repeated content that aligns with the audience’s values, lifestyle, or cultural background can reinforce this trust, deepening influence and shaping long-term attitudes [54-56]. However, this same trust dynamic can become problematic when influencers lack scientific training, do not disclose commercial ties, or prioritise engagement over accuracy [54-56]. Studies have shown that followers may not distinguish between health advice given for public-health reasons and advice driven by marketing or personal belief, which can blur the line between education and entertainment [54-56]. When trust is misused or information is misleading, entire communities can internalise inaccurate health beliefs that are difficult to correct through official channels alone [54-56]. From a public‑health perspective, understanding these trust dynamics is, therefore, essential for both collaborating with influencers who promote evidence‑based messages and developing strategies to counter harmful narratives that exploit followers’ loyalty [54-56].

Conflicts can arise when commercial interests intersect with public health messaging in influencer content, creating tension between marketing goals and population‑level health outcomes [54,57,58]. Some health‑related products, such as supplements, detox regimens, or cosmetic treatments, are promoted by influencers in ways that downplay risks, exaggerate benefits, or imply medical authority without clear evidence [54,57,58]. In these cases, the primary objective may be sales or brand visibility rather than accurate health information, which can lead to public misunderstanding and, in some cases, unsafe behaviours [54,57,58]. When such content reaches large audiences quickly, it can compete directly with public‑health campaigns that emphasise moderation, safety, and evidence‑based practice [54,57,58]. Studies have also shown that when influencers receive payment, free products, or other incentives, their audiences may not always recognise these relationships, which can blur the boundary between independent advice and branded promotion [54,57,58]. This lack of transparency can erode trust in both influencers and health‑related content more broadly, especially once problematic claims are exposed [54,57,58]. From a public health standpoint, these commercial pressures highlight the need for clearer guidelines about how influencers communicate health information, including requirements to disclose sponsorships, distinguish opinion from evidence, and avoid promoting products that conflict with established public health recommendations [54,57,58]. Addressing these conflicts does not mean banning commercial content entirely, but rather creating a framework in which profit‑driven messaging cannot override accurate, responsible health communication [54,57,58].

Several case examples show how influencer‑led campaigns have been used to promote public health messages, including vaccination, screening, and mental health awareness [55,57,59]. In some settings, public health agencies or non‑profit organisations have partnered with influencers to share short, relatable videos or posts that normalise behaviours such as getting tested, seeking help for mental health concerns, or participating in vaccination drives [55,57,59]. These campaigns often rely on storytelling, hashtags, and challenges that encourage followers to share their own experiences, creating a sense of community and mutual support [55, 57, 59]. In certain evaluations, such initiatives have been associated with increased awareness, higher self‑reported willingness to act, and, in some cases, measurable rises in service uptake among targeted groups [55,57,59]. These examples also reveal that the effectiveness of influencer‑led campaigns depends heavily on how they are designed and implemented [55,57,59]. When influencers are carefully selected, provided with clear evidence‑based messages, and given time to integrate those messages into their usual style, audiences are more likely to receive them as natural and credible rather than forced or promotional [55,57,59]. Conversely, when partnerships feel inauthentic, rushed, or poorly aligned with the influencer’s existing audience, engagement may be low and the impact on behaviour weaker [55,57,59]. Overall, these case studies suggest that influencer‑led campaigns can be a valuable addition to public‑health toolkits, but they work best when they are collaborative, well‑resourced, and grounded in the same communication principles that guide more traditional health‑promotion efforts [55,57,59].

Influencers can also become powerful channels for spreading health misinformation, especially when their content is emotionally charged, visually compelling, or difficult for non‑experts to critically evaluate [54,56,58]. Because influencers often speak in casual, personal tones, audiences may not recognise that claims about vaccines, treatments, or diagnostic tests lack scientific backing, even when those claims contradict official guidance [54,56,58]. In some documented cases, a relatively small number of influential accounts have helped disseminate false narratives about side effects, conspiracy theories, or unproven remedies, leading to confusion, delayed care, and reduced uptake of evidence‑based interventions [54,56,58]. The risk is amplified by the way platforms prioritise engagement, which can reward dramatic or polarising content over balanced, nuanced information [54,56,58]. When misinformation from influencers goes viral, public health agencies may struggle to respond quickly enough, and their corrections may not reach the same audiences or carry the same perceived authenticity [54,56,58]. In addition, followers may be more inclined to trust an influencer they have followed for years than to accept a one‑off correction from an unfamiliar government account, which can make it particularly difficult to reverse the effects of widely shared falsehoods [54,56,58]. From a policy perspective, these risks underline the need for strategies that support credible influencers, improve media literacy, and strengthen mechanisms for identifying and mitigating harmful health content before it spreads widely [54,56,58].

Online Communities and Peer‑to‑Peer Health Communication

Health‑related support groups have proliferated on social media platforms, bringing together people who share similar conditions, concerns, or treatment experiences [57,60,61]. These groups often form organically when individuals post about their challenges and others respond with empathy, advice, or personal stories, gradually building a network of mutual support [57,60,61]. Over time, members establish shared rules for interaction, nominate administrators, and create spaces where sensitive topics can be discussed openly away from mainstream feeds [57,60,61]. In some cases, these communities focus on specific diseases like diabetes, cancer, or mental health issues, while others address broader themes such as parenting or chronic pain management [58,62]. What makes these online groups effective is their ability to provide a sense of belonging and continuity for people who may feel isolated in their daily lives [57,60,61]. Members can connect at any hour, share updates on their progress or setbacks, and receive immediate encouragement or practical suggestions from peers who understand their situation firsthand [57,60,61]. At the same time, the formation process is not always smooth, as groups must navigate conflicts, differing opinions, and the risk of unhelpful advice [57,61]. Nevertheless, the literature shows that these digital support networks can fill gaps left by formal health services, particularly when people need emotional reinforcement or information that feels more relevant than generic public health guidance [57,60,61].

Peers within online communities can exert considerable influence on whether and how health behaviours are adopted [50,60,61]. Members often look to others in the group as informal role models, observing what they do, how they manage symptoms, or what strategies they use to cope with treatment side effects [50,60,61]. When someone shares a success story, such as sticking to a new diet, attending regular check‑ups, or trying a recommended supplement, others may feel encouraged to try similar approaches, especially if the person seems relatable or credible [50,60,61]. This process of imitation is strengthened when group members provide positive feedback, share their own results, or normalise the behaviour as something achievable in everyday life [50,60,61]. Research shows that peer influence tends to be strongest when the behaviour is seen as socially desirable within the community and when people feel a sense of shared identity or mutual accountability [50,60,61]. For example, if a group consistently celebrates those who follow exercise routines or medication schedules, new members may adopt these practices to gain approval and belonging [50,61]. At the same time, peer influence can reinforce unhelpful habits if the group prioritises short‑term comfort over long‑term health benefits [50,60,61]. Either way, these dynamics demonstrate that online communities shape behaviour not through formal authority, but through the subtle power of example, encouragement, and social reinforcement among equals [50,60,61].

Community norms within online health groups play a central role in defining which health practices are encouraged, tolerated, or discouraged [57,60,61]. Norms emerge gradually as members post about what works for them, critique approaches they find unhelpful, and affirm behaviours that align with the group's shared values [50,60,61]. Over time, these patterns create unwritten rules about what is normal or expected, such as adhering to particular diets, prioritising certain treatments, or avoiding mainstream medical advice [50,60,61]. New members learn these norms by observing discussions, receiving direct feedback, and gradually participating in the conversation [57,60,61]. Studies show that norms can both promote and constrain health practices [57,60,61]. On one hand, groups that emphasise evidence‑based behaviours like regular screening or medication adherence can help members maintain healthy routines that they might otherwise neglect [60,61]. On the other hand, norms that favor unproven remedies or discourage professional consultation can lead to risky decisions, especially if the group becomes an echo chamber where alternative views are dismissed [57,60,61]. The strength of these norms depends on group size, leadership, and how much members identify with the community [57, 60, 61]. In short, community norms act as a social glue that guides individual behaviour, making online health groups powerful spaces for either reinforcing or challenging established health practices [57,60,61].

Online health communities offer both emotional and informational support that can help people navigate illness and treatment [55,57,60]. Emotional support comes through expressions of empathy, encouragement, and validation from peers who have faced similar struggles, helping to reduce feelings of isolation or despair [55,57,60]. Members often share how they cope with pain, fatigue, or uncertainty, offering reassurance that these experiences are common and manageable [55,57,60]. Informational support takes the form of practical advice about managing symptoms, finding resources, or dealing with side effects, often drawn from personal experience rather than textbooks [55,57,60]. Together, these forms of support create a space where people can ask questions, vent frustrations, and find strategies that feel relevant to their lives [55,57,60]. Research suggests that this dual support can improve quality of life, adherence to treatment, and even clinical outcomes in some cases [55,57,60]. People who participate actively in supportive online spaces often report feeling more hopeful and equipped to handle their health challenges [55,60]. At the same time, the line between helpful support and unverified advice can be thin, as emotional connections may lead members to endorse information that has not been rigorously tested [55,57,60]. Still, for many, the combination of emotional reassurance and peer wisdom fills a gap that formal health services cannot always address, making digital communities valuable allies in the journey toward better health [55,57,60].

Online health communities can be powerful conduits for both accurate health information and misinformation, depending on what members share and endorse [50,54,61]. Accurate information spreads when people post links to reliable sources, describe evidence‑based practices, or highlight successful outcomes from recommended treatments [50,54,61]. In supportive groups, this knowledge exchange helps members make informed choices about screening, vaccination, or lifestyle changes, often in a more accessible format than official materials [50,54,61]. However, the same mechanisms that amplify helpful content can also propagate unverified claims, personal anecdotes taken as general truth, or outright false narratives that gain traction through repetition and emotional appeal [50,54,61]. Studies show that misinformation thrives when it aligns with group beliefs, addresses felt needs, or comes from trusted members, while accurate information may struggle if it contradicts community norms or lacks personal resonance [50,54,61]. Moderators can play a role in curbing false claims, but their influence is limited if the group culture prioritises individual stories over external validation [50,54,61]. This dual nature means that communities are neither inherently good nor bad for public health; their impact depends on the balance between reliable and unreliable content circulating within them [50,54,61].

The spread of health misinformation on social media platforms and its public health implications

Mechanisms and Drivers of Health Misinformation

Social media algorithms play a key role in amplifying health misinformation by prioritising content that generates high engagement, regardless of its accuracy [62-64]. These algorithms rank posts based on likes, shares, comments, and time spent viewing, which often favours sensational, emotional, or controversial claims about vaccines, treatments, or health risks [62-64]. As a result, misinformation spreads faster and wider than balanced, evidence‑based information, because it triggers strong reactions that keep users scrolling and interacting [62-64]. Over time, this creates feedback loops where the most provocative health content rises to the top of feeds and recommendations, exposing larger audiences to potentially harmful ideas [62-64]. Researchers have shown that this amplification is not intentional but emerges from the design of platforms that optimise for user attention rather than public‑health outcomes [62-64]. For example, during health emergencies, posts questioning official guidance or promising miracle cures often receive more visibility than posts sharing clinical evidence or public‑health recommendations [62-64]. The effect is particularly pronounced for topics that evoke fear, anger, or hope, which are common in health misinformation [62-64]. In response, some studies have called for algorithm tweaks that promote credible sources, but the commercial incentives of platforms make such changes difficult to implement [62-64]. Ultimately, algorithmic amplification turns misinformation into a structural feature of social media rather than just an occasional problem, with consequences for public trust and health behaviour [62-64].

Bots and coordinated campaigns have become significant drivers of health misinformation on social media, artificially inflating the visibility of false or misleading claims [62,64,65]. Automated accounts, often controlled by networks of actors, can generate thousands of posts, likes, and shares in short periods, making it appear that a particular health narrative has widespread support [62,64,65]. These operations often target specific topics such as vaccine safety, pandemic origins, or alternative treatments, using repetitive messaging and hashtags to dominate conversations and push content into trending topics [62,64,65]. By mimicking human behaviour, bots can evade detection while amplifying claims that would otherwise remain marginal [62,64,65]. Studies have documented how coordinated campaigns exploit platform mechanics to create the illusion of consensus, making it harder for users to distinguish genuine discussion from manufactured outrage [62,64,65]. In some cases, these efforts have been linked to state actors, commercial interests, or ideological groups seeking to influence public opinion during health crises [62,64,65]. The result is often a distorted picture of public sentiment that undermines trust in health authorities and discourages recommended behaviours [62,64,65]. While platform companies have developed tools to detect and remove bot activity, the sophistication of these campaigns continues to outpace detection, and the sheer volume of content makes complete removal difficult [62,64,65]. From a public‑health perspective, understanding the role of bots highlights the need for better monitoring, rapid response, and public education about how online conversations can be manipulated [62,64,65].

Cognitive biases make people more susceptible to health misinformation on social media, shaping how they perceive, interpret, and share false information [62,63,66]. Confirmation bias leads users to pay more attention to claims that align with existing beliefs, such as vaccine dangers or miracle cures, while dismissing contradictory evidence [62,63,66]. Availability bias then reinforces these views when sensational content is constantly shown in feeds, making rare risks seem common and simple solutions seem plausible [62,63,66]. In this way, biases turn ordinary scrolling into a process of selective exposure that strengthens misconceptions over time [62,63,66]. Research has also shown that anchoring bias can make the first health claim a user encounters seem more credible than subsequent corrections, especially if it comes from a familiar source or appears in a trusted context [62,63,66]. Emotional biases further complicate matters, as fear‑inducing or hope‑promising misinformation often feels more compelling than dry, factual information, prompting shares without verification [62,63,66]. These patterns explain why debunking misinformation can sometimes backfire, because repeating a false claim-even to refute it-can inadvertently strengthen its familiarity [62,63,66]. Public health communicators therefore need to anticipate these biases when designing messages, using strategies such as pre‑emptive inoculation, relatable narratives, and clear visuals that appeal to both reason and emotion [62,63,66]. Understanding cognitive biases helps explain why misinformation persists despite fact‑checking and correction, and points to the need for interventions that work with, rather than against, the way people process information online [62,63,66].

False health information tends to spread more quickly and widely than accurate information on social media, due to differences in emotional appeal and platform mechanics [64,65,67]. Studies comparing the diffusion patterns of verified facts and debunked claims have found that misinformation often reaches more people faster, because it evokes stronger reactions such as fear, anger, or outrage that prompt immediate sharing [64,65,67]. In contrast, accurate information about vaccines, treatments, or prevention measures is typically more technical and less emotionally charged, so it generates lower engagement and slower diffusion [64,65,67]. This disparity means that by the time public health agencies respond with corrections, the false narrative may already have reached a wide audience [64,65,67]. Virality is also shaped by network effects, where content spreads through dense clusters of like‑minded users who reinforce each other’s beliefs [64,65,67]. Misinformation thrives in these environments, while accurate information may struggle to cross group boundaries or gain traction in sceptical communities [64,65,67]. Over time, repeated exposure to viral falsehoods can normalise them, making them seem like common knowledge even when contradicted by evidence [64,65,67]. Researchers have therefore called for changes in how platforms handle health content, including faster moderation of high‑risk misinformation and better promotion of credible sources, though implementation remains uneven [64,65,67]. The persistent advantage of false information in virality dynamics poses a structural challenge for public health communication, requiring proactive strategies that compete on emotional and social terms as much as on factual accuracy [64,65,67].

Political and cultural factors significantly shape how health misinformation spreads and is received on social media [63,66,68]. In politically polarised environments, health claims often become proxies for larger ideological battles, with misinformation about vaccines, masks, or pandemic origins aligning with partisan identities [63,66,68]. Users are more likely to share and believe information that reinforces their political tribe, even when it lacks evidence, because it signals loyalty and validates group beliefs [63,66,68]. Cultural norms further colour these dynamics, as different societies have varying levels of trust in institutions, tolerance for uncertainty, and expectations about personal versus collective responsibility [63,66,68]. For example, in settings where distrust of government or science is high, misinformation framing health measures as control mechanisms can gain traction more easily than in contexts where institutional authority is accepted [63,66,68]. Similarly, cultural attitudes toward risk, fate, or traditional remedies can make certain false claims more plausible to particular groups, leading to persistent pockets of resistance to public‑health recommendations [63,66,68]. These factors create environments where misinformation is not just an information problem but a deeply rooted social and political phenomenon that requires tailored responses rather than universal corrections [63,66,68]. Public health communication must therefore navigate these divides carefully, using culturally sensitive messengers, locally relevant examples, and messages that respect underlying values while gently challenging falsehoods [63,66,68].

Public Health Consequences of Misinformation

Misinformation about vaccines circulating on social media has been strongly linked to increased vaccine hesitancy and outright refusal, creating significant public health challenges [19,67,68]. False claims about side effects, ineffectiveness, or hidden agendas have convinced some parents and individuals to delay or avoid vaccination, even when official data show vaccines are safe and effective [19,67,68]. These narratives often spread through emotionally charged stories, selective statistics, or distrust in pharmaceutical companies and governments, which resonate with people who already feel uncertain or marginalised [19,67,68]. In some communities, this has led to lower vaccination coverage, leaving pockets of unvaccinated people vulnerable to outbreaks of preventable diseases [19,67,68]. Studies have found that exposure to anti‑vaccine content online can shift attitudes quickly, especially among those who spend more time on social media or encounter it through trusted contacts [19,67,68]. Even one compelling post or video can plant doubt that is hard to dispel with facts alone, because fear and distrust are more emotionally engaging than reassurance [19,67,68]. Public health teams therefore face an uphill battle to rebuild confidence through transparent communication, community engagement, and counter‑messaging that addresses specific fears without dismissing legitimate concerns [19,68,69]. The pattern is clear: misinformation does not just create confusion; it translates into measurable drops in vaccination uptake that threaten herd immunity and population‑level protection [19,67,68].

Social media has also facilitated the spread of harmful health practices, often presented as safe alternatives or quick fixes [63,65,67]. Posts promoting unproven detox regimens, extreme diets, or DIY treatments for chronic conditions can gain millions of views, encouraging people to try methods that lack scientific support and may cause physical harm [63,65,67]. Influencers or wellness accounts frequently share before‑and‑after photos, personal testimonials, or simple recipes that promise dramatic results, which can appeal to those frustrated with conventional medicine or seeking control over their health [63,65,67]. When followers adopt these practices, some experience adverse effects such as nutritional deficiencies, interactions with prescribed medications, or delayed treatment for serious conditions [63,65,67]. The problem is compounded by the fact that harmful content often circulates in closed groups or algorithmic bubbles where challenges to it are rare or invisible [63,65,67]. Public health implications include increased emergency visits, worsened health outcomes, and a growing distrust in evidence‑based care as people blame doctors or hospitals for their experiences with unproven remedies [63,65,67]. Over time, this creates a feedback loop where more people turn away from proven interventions toward whatever seems to work in the stories they see online [63,65,67].

Repeated exposure to health misinformation on social media has contributed to a broader erosion of trust in health institutions, making it harder for public health authorities to communicate effectively [62,64,65]. When users encounter claims that doctors, hospitals, or governments are hiding information, prioritising profit, or colluding with industry, these narratives can create a pervasive sense that official sources cannot be relied upon [62,64,65]. Over time, this distrust shifts how people evaluate health information, making them more likely to favour personal stories, alternative experts, or crowd wisdom over institutional guidance [62,64,65]. The consequences are far‑reaching [62,64,65]. Once trust is undermined, even accurate public‑health messages about vaccination, screening, or lifestyle changes may be dismissed as part of a larger conspiracy, reducing their impact [62,64,65]. In some cases, people actively avoid recommended services because they believe the system is corrupt or incompetent, which can worsen health disparities and delay outbreak responses [62,64,65]. Rebuilding trust requires more than fact‑checking; it demands sustained, transparent communication that acknowledges past mistakes, engages communities, and shows that health institutions are accountable and responsive to public concerns [62,64,65]. Without addressing this erosion, misinformation will continue to weaken the foundation of collective health protection [62,64,65].

Health misinformation on social media can directly contribute to increased disease transmission by discouraging protective behaviours and promoting risky ones [19,63,67]. False claims that masks do not work, that social distancing is unnecessary, or that certain infections are harmless have led some people to ignore public‑health guidance during outbreaks, increasing the likelihood of spread [19,63,67]. These messages often gain traction because they promise freedom or normalcy, appealing to those who feel burdened by restrictions or skeptical of authority [19,63,67]. When widely shared, they can normalise behaviours that accelerate transmission, such as large gatherings or non‑compliance with quarantine [19,63,67]. Evidence from several outbreaks shows that areas with high exposure to such content have sometimes experienced faster rises in cases or lower adherence to preventive measures [19,63,67]. In extreme cases, misinformation has delayed testing, treatment, or isolation, allowing infections to spread silently through communities before they are detected [19,63,67]. The public health impact is clear: when protective actions are undermined by widely circulated falsehoods, the result is higher morbidity, mortality, and strain on health systems [19,63,67]. Rapid, credible counter‑messaging is essential, but it must compete with content that travels faster and feels more immediate [19,63,67].

The COVID‑19 pandemic provides stark case studies of how health misinformation, or the “infodemic,” can overwhelm public health responses and shape outbreak trajectories [19,67,69]. Early in the pandemic, claims about unproven treatments, mask ineffectiveness, and vaccine dangers spread rapidly across platforms, creating confusion and undermining compliance with basic preventive measures [19,67,69]. In some countries, these narratives contributed to delayed lockdowns, uneven mask use, and vaccine hesitancy that prolonged transmission and increased mortality [19,67,69]. Public health agencies struggled to keep pace with the volume and speed of false information, which often came from seemingly credible sources or was amplified by algorithms [19,68,70]. Later phases of the pandemic showed similar patterns around boosters and variants, where misinformation about side effects or conspiracy theories kept vaccination rates below the thresholds needed for sustained control [19,67,69]. These cases illustrate how an infodemic can interact with an epidemic, turning what could have been straightforward public health measures into contested, polarised debates [19,67,69]. The COVID‑19 experience has left a legacy of lessons about the need for proactive monitoring, rapid response teams, and communication strategies that can cut through the noise of misinformation during crises [19,67,69].

Strategies for detecting, managing, and countering health misinformation on social media

Technological and Algorithmic Interventions

AI has become a primary tool for detecting health misinformation on social media, using machine learning to identify patterns in text, images, and user behaviour that signal falsehoods [70-72]. These systems are trained on large datasets of verified true and false claims about vaccines, treatments, and public‑health measures, learning to recognise linguistic cues, source credibility, and propagation patterns that distinguish reliable content from deceptive content [70-72]. Once deployed, AI classifiers can scan posts in real time, flagging suspicious material for human review or automatic action, which allows platforms to respond faster than manual moderation alone [70-72]. Studies evaluating these approaches have shown that well‑trained models can achieve high accuracy on common types of health misinformation, such as anti‑vaccine claims or fake remedy promotions [70-72]. However, performance varies by language, topic, and context, and sophisticated falsehoods that mimic legitimate discourse can still evade detection [70-72]. In addition, AI systems require constant retraining to keep pace with evolving tactics and new health emergencies [70-72]. Despite these challenges, AI‑based detection represents a scalable first line of defence, enabling platforms to prioritise harmful content for intervention while allowing accurate information to circulate more freely [70-72].

Platform policies for removing health misinformation have evolved in response to public‑health crises, balancing free speech with the need to protect users from harmful content [73-75]. These policies typically define prohibited content, such as false claims about vaccines, treatments, or pandemic measures, and outline procedures for detection, review, and action [73-75]. Once flagged-often through AI, user reports, or expert partnerships-posts may be removed, accounts suspended, or visibility reduced, depending on severity and context [73-75]. Platforms have also introduced labels, warning notices, and reduced distribution for borderline content that does not meet the removal threshold [73-75]. Research on these policies suggests that proactive removal can limit the reach of dangerous misinformation, particularly when applied consistently and transparently [73-75]. However, enforcement is uneven, as cultural differences, legal requirements, and resource constraints affect how rules are applied across regions and languages [73-75]. Critics also argue that removal can backfire by reinforcing beliefs that platforms are censoring dissenting views, which may drive users to alternative spaces [73-75]. From a public‑health perspective, effective policies require clear criteria, rapid response, and collaboration with health authorities, while avoiding overreach that undermines trust in the moderation process itself [73-75].

Fact‑checking systems have emerged as a key strategy for countering health misinformation, verifying claims against reliable sources and providing users with clear assessments of accuracy [70,72,76]. These systems often involve independent organisations or platform‑integrated tools that investigate specific claims about vaccines, treatments, or public‑health measures, then publish ratings such as “true,” “false,” or “mixture” [70,72,76]. When integrated with social media, fact‑checks can be linked to or displayed alongside disputed posts, giving users immediate context without removing the content entirely [70,72,76]. Studies evaluating fact‑checking have found that it can reduce belief in and sharing of misinformation, particularly when checks are timely, concise, and come from trusted sources [70,72,76]. However, effectiveness depends on how widely the corrections reach, whether users engage with them, and how they interpret the information [70,72,76]. In some cases, fact‑checks have little impact on committed believers or may even reinforce misconceptions by repeating the original false claim [70,72,76]. To address this, researchers have advocated for pre‑emptive fact‑checking, crowd‑sourced verification, and formats that emphasise evidence over confrontation [70,72,76]. While not a complete solution, fact‑checking systems provide a transparent, evidence‑based way to challenge health misinformation at scale, complementing other interventions such as education and platform moderation [70,72,76].

Content moderation strategies for health misinformation combine automated detection, human review, and user reporting to identify and address harmful posts efficiently [73-75]. Moderation begins with AI filters that scan for keywords, patterns, or sources associated with falsehoods about vaccines, treatments, or public‑health guidance, flagging high‑risk content for closer examination [73-75]. Trained human reviewers then assess context, intent, and potential impact, deciding whether to remove, label, demote, or leave the post intact [73-75]. User reports provide an additional layer, allowing communities to surface emerging issues that algorithms might miss [73-75]. Research on these strategies shows that tiered approaches-escalating from warnings to removal based on severity-can reduce the spread of dangerous misinformation while preserving open discussion [73-75]. However, challenges persist in scaling moderation across languages, cultures, and rapidly evolving health topics, and over‑moderation can alienate users or drive them to less regulated platforms [73-75]. Effective strategies also require clear guidelines, transparency about decisions, and collaboration with health experts to ensure that moderation aligns with public‑health priorities rather than arbitrary or inconsistent standards [73-75]. Overall, content moderation remains an imperfect but essential tool for managing health misinformation, balancing the need to protect public safety with the value of free expression [73-75].

Automated detection systems for health misinformation have clear limitations that constrain their effectiveness as a standalone solution [64,70,76]. Despite advances in machine learning, these systems struggle with context, nuance, and novel misinformation that does not match training data, such as satire, sarcasm, or emerging health claims [64,70,76]. False positives can also occur, flagging legitimate discussions or minority views as misinformation, which erodes user trust and creates perceptions of censorship [64,70,76]. Language barriers further complicate detection, as most systems perform best on widely used languages and falter on dialects, slang, or low‑resource languages where health misinformation may thrive [64,70,76]. Studies have documented these issues in real‑world applications, where automated tools missed sophisticated falsehoods or over‑corrected ambiguous content, leading to both under‑ and over‑moderation [64,70,76]. The rapid evolution of tactics used by misinformation spreaders also requires constant retraining, which is resource‑intensive and can lag behind new developments [64,70,76]. Moreover, AI systems may inherit biases from their training data, systematically underperforming for certain topics, regions, or communities [64,70,76]. These limitations mean that automated detection works best as part of a broader strategy that includes human oversight, user education, and proactive public‑health communication, rather than as a complete fix for the complex problem of health misinformation [64,70,76].

Public Health Communication and Digital Literacy Strategies

Educational campaigns to improve media literacy have become a cornerstone of efforts to counter health misinformation on social media [73,75,77]. These campaigns teach people how to evaluate online health information by checking sources, identifying bias, recognising emotional manipulation, and cross‑referencing claims with credible outlets [73,75,77]. Simple tools like checklists, infographics, and short videos help users spot red flags such as anecdotal evidence, exaggerated promises, or lack of transparency about funding [73,75,77]. By building these skills, public health communicators aim to create communities that are more sceptical of dubious claims and better equipped to share accurate information [73,75,77]. Studies evaluating these initiatives have shown that targeted media literacy programmes can improve people’s ability to distinguish fact from fiction, especially when delivered through schools, workplaces, or community groups [73,75,77]. For example, participants exposed to brief training about source credibility and logical fallacies have been less likely to share or believe misleading health posts in follow‑up tests [73,75,77]. However, challenges remain: not everyone has the time, interest, or prior knowledge to engage deeply with these materials, and skills learned in one context may not transfer to others [73,75,77]. Despite these limitations, media literacy campaigns represent a proactive, empowering approach that addresses the root causes of misinformation susceptibility rather than simply reacting to individual false posts [73,75,77].

Trusted institutions such as public health agencies, universities, and professional bodies play a vital role in countering health misinformation through authoritative, timely messaging [19,71,77]. These organisations have the expertise and credibility to provide evidence‑based clarifications that can reach broad audiences and restore confidence in recommended health practices [19,71,77]. By issuing clear, accessible statements through their own channels and partnering with platforms, they can amplify accurate information and directly address circulating falsehoods [19,71,77]. The key is to respond quickly, use plain language, and acknowledge legitimate concerns while firmly correcting errors [19,71,77]. Research has shown that when institutions engage proactively rather than reactively, they can limit the spread of misinformation and maintain public trust, especially during crises [19,71,77]. For example, official accounts posting infographics, live sessions, or myth‑busting threads have sometimes outperformed viral falsehoods in reach and engagement, particularly when they use relatable formats and credible spokespeople [19,71,77]. However, institutional messaging can struggle against deeply held beliefs or distrust of authority, and overly defensive or technical responses may be dismissed as biased or elitist [19,71,77]. To be effective, counter‑messaging from trusted institutions must balance authority with empathy, transparency, and accessibility, while building long‑term relationships with online communities rather than treating each incident in isolation [19,71,77].

Community engagement approaches to countering health misinformation emphasise working with rather than talking at online groups, building relationships and shared understanding [74,75,77]. These strategies involve identifying key influencers, discussion spaces, and cultural touchpoints where health conversations happen naturally, then participating in those conversations with respect and genuine interest [74,75,77]. Rather than posting generic corrections, public health actors might ask questions, share stories, or collaborate on content that addresses local concerns while gently introducing evidence [74,75,77]. Over time, this builds trust and makes people more open to accurate information [74,75,77]. Evaluations of community‑based interventions have found that sustained, dialogue‑oriented engagement can reduce the uptake of misinformation and increase willingness to follow public‑health guidance, particularly in tight‑knit or sceptical groups [74,75,77]. For example, working with local leaders or popular accounts to co‑create messages about vaccination or testing has sometimes led to higher acceptance than top‑down campaigns alone [74,75,77]. However, these approaches require time, cultural sensitivity, and the ability to listen as much as to speak, and they may not work in highly polarised or anonymous spaces [74,75,77]. When done well, though, community engagement turns misinformation countering into a collaborative process that strengthens social ties and public‑health resilience [74,75,77].

Collaboration between public health organisations and social media platforms has become a key strategy for detecting and managing health misinformation at scale [71-73]. Platforms can prioritise credible sources, label questionable content, or temporarily reduce the visibility of posts that violate health‑related guidelines, while health agencies provide expertise on what constitutes harmful misinformation [71-73]. These partnerships often involve shared dashboards, rapid reporting channels, and co‑developed policies that balance free speech with public safety [71-73]. During crises, such collaborations have enabled faster identification and removal of dangerous claims about treatments or vaccines [71-73]. Studies of these efforts have shown mixed results: some interventions have successfully limited the reach of misinformation, but others have been criticised for inconsistent enforcement or unintended suppression of legitimate debate [71-73]. Platforms face commercial pressures to maintain user engagement, while health agencies worry about over‑reach or bias, making trust and clear agreements essential [71-73]. Despite challenges, collaboration offers a practical way to leverage platform tools and data for public‑health benefit, though it requires ongoing dialogue, transparency about decision‑making, and evaluation of impacts on both misinformation and public discourse [71-73].

Evaluating the effectiveness of misinformation interventions requires careful measurement of both immediate and longer‑term outcomes [71,76,77]. Studies often use metrics such as changes in sharing rates, belief in specific claims, or self‑reported media literacy before and after exposure to educational content, corrections, or platform changes [71,76,77]. Some have tracked how quickly false posts lose visibility after moderation or whether counter‑messages reach the same audiences as the original misinformation [71,76,77]. More sophisticated evaluations link digital interventions to real‑world behaviours, such as vaccination uptake or healthcare utilisation, though establishing causality remains difficult [71,76,77]. Findings are encouraging but varied: media literacy campaigns can improve critical thinking, corrections from trusted sources can reduce belief in falsehoods, and platform actions can limit spread, but effects are often modest and context‑specific [71,76,77]. Challenges include the difficulty of reaching entrenched believers, the risk of backfire effects, and the resource intensity of sustained monitoring [71,76,77]. Rigorous evaluation is therefore essential to identify what works, for whom, and under what conditions, ensuring that public‑health efforts are evidence‑based rather than driven by assumptions or short‑term pressures [71,76,77].

Ethical, legal, and privacy considerations in the use of social media data for public health

Ethical Issues in Data Collection and Surveillance

Obtaining informed consent for social media data use in public health surveillance presents unique challenges, because users typically post content without anticipating that it will be analysed for epidemiological purposes [78-80]. Most platforms' terms of service grant broad data‑access rights, but these agreements do not specifically address health research or surveillance, leaving users unaware that their posts about symptoms or health concerns could contribute to population‑level monitoring [78-80]. This raises questions about whether passive data collection respects the autonomy and privacy expectations of individuals who view social media as a personal space [78-80]. Researchers have argued that retroactive consent is impractical for large‑scale surveillance, and that anonymisation alone may not be sufficient to protect identities in the era of sophisticated re‑identification techniques [78-80]. In some cases, users might support public‑health uses of their data if asked directly, but without clear opt‑in mechanisms, the default becomes using data without explicit permission [78-80]. This tension underscores the need for transparent policies, user controls, and communication about how social media data are handled in health contexts, so that surveillance benefits do not come at the cost of eroding trust or violating reasonable privacy expectations [78-80]. Balancing these ethical concerns requires ongoing dialogue between researchers, platforms, and the public about what constitutes acceptable use of voluntarily shared digital traces [78-80].

Passive data collection from social media, gathering posts, profiles, and interactions without direct user involvement, raises significant ethical concerns about privacy, autonomy, and potential harm [79-81]. Unlike active studies where participants know they are being observed, passive surveillance treats public posts as research material, often without notifying or seeking permission from those who created them [79-81]. This approach can inadvertently capture sensitive health details that users did not intend to share widely or with researchers, raising risks of stigma, discrimination, or unwanted exposure if data are de‑anonymised or misused [79-81]. Literature on these issues emphasises that the public nature of social media does not eliminate ethical obligations, and that researchers must consider the context in which data were posted and the reasonable expectations of privacy [79-81]. For example, a casual mention of symptoms might be fine for personal sharing but problematic when aggregated into surveillance profiles that could be linked back to individuals [79-81]. Critics also worry about the power imbalance between data collectors and subjects, and the potential for passive collection to normalise constant monitoring without adequate safeguards [79-81]. Ethical practice therefore demands robust anonymisation, minimal data retention, and institutional review processes that weigh public‑health benefits against individual rights, ensuring that passive surveillance does not become an unchecked expansion of data extraction from unsuspecting users [79-81].

The use of social media for public health surveillance carries risks of overreach, where monitoring intended for disease detection expands into broader behavioural tracking or social control [17,78,82]. Without clear boundaries, agencies might use health‑related data to profile communities, predict behaviours, or justify interventions beyond immediate public‑health needs [17,78,82]. This can erode civil liberties, create chilling effects on free speech, and disproportionately affect marginalised groups already subject to heightened scrutiny [17,78,82]. Studies have highlighted how surveillance systems, once established, tend to grow in scope, collecting more data types, retaining information longer, and sharing it across agencies or borders [17,78,82]. In health contexts, vague definitions of “public interest” can justify expansive data use, while technical capabilities outpace legal and ethical frameworks [17,78,82]. For example, symptom tracking could evolve into monitoring political dissent or social movements if health data are repurposed [17,78,82]. To mitigate overreach, researchers advocate for strict mandates, independent oversight, data‑use audits, and sunset clauses that limit retention and purpose, ensuring surveillance remains focused on legitimate public‑health goals rather than creeping into general population control [17,78,82].

Balancing the public‑health benefits of social media surveillance with individual rights requires careful ethical deliberation and practical safeguards [78,79,83]. On one hand, aggregated data can enable early outbreak detection, targeted interventions, and resource allocation that protect entire communities from harm [78,79,83]. On the other hand, individual privacy, autonomy, and freedom from unwarranted intrusion must be protected, especially since data subjects rarely consent to such uses [78,79,83]. This tension is particularly acute when surveillance disproportionately affects vulnerable groups or when benefits are uncertain while risks are immediate [78,79,83]. Literature calls for proportionality: data collection should be necessary, minimal, and time‑limited, with clear justification showing that public good outweighs private costs [78,79,83]. Mechanisms such as data minimisation, strong anonymisation, independent ethics review, and public reporting help ensure accountability and rebuild trust [78,79,83]. Some frameworks propose participatory governance, where communities help define acceptable uses and monitor compliance [78,79,83]. Ultimately, effective balance requires ongoing evaluation, transparent decision‑making, and recognition that surveillance is a social contract, not an absolute right, demanding constant justification to those whose data enable public‑health gains [78,79,83].

Ethical frameworks for digital epidemiology aim to guide the responsible use of social media data while maximising public‑health benefits [17,79,84]. These frameworks typically emphasise principles such as beneficence, non‑maleficence, justice, respect for persons, transparency, and accountability [17,79,84]. They provide structured ways to assess whether proposed surveillance activities are proportionate, necessary, and respectful of rights, often through checklists, decision trees, or institutional review processes [17,79,84]. For instance, frameworks may require justification for data collection, plans for minimisation and anonymisation, and strategies for community engagement [17,79,84]. Applied examples show these tools helping researchers navigate consent challenges, privacy risks, and equity concerns in real projects [17,79,84]. However, implementation varies, as frameworks are often advisory rather than mandatory, and global differences in regulation create gaps [17,79,84]. Advocates call for stronger international standards, training for practitioners, and integration with legal requirements like GDPR or HIPAA [17,79,84]. By providing principled guidance, ethical frameworks help ensure digital epidemiology serves public health without compromising core values or eroding trust in the systems that depend on voluntary data sharing [17,79,84].

Legal and Regulatory Frameworks Governing Social Media Data Use

Data protection laws like the General Data Protection Regulation (GDPR) in Europe and similar frameworks elsewhere have become central to governing how social media data can be used for public health purposes [78,79,82]. These laws require that personal data, including health‑related posts, user profiles, and location information, be collected and processed only with clear consent, transparent purpose, and robust security measures [78,79,82]. Public health researchers must demonstrate that their use of social media data serves a legitimate public interest while minimising risks to individual privacy, often through techniques like data anonymisation, aggregation, and time‑limited access [78,79,82]. In practice, GDPR and its equivalents create both opportunities and constraints: they protect users from exploitation but can complicate large‑scale surveillance projects that rely on real‑time, granular data [78,79,82]. For example, scraping public posts for outbreak monitoring may be permissible under public‑health exemptions, but linking those posts to identifiable users or using them for predictive modelling often requires formal approval and safeguards [78,79,82]. Non‑compliance can lead to heavy fines and loss of public trust, making legal review essential for any social‑media-based health research [78,79,82]. These frameworks therefore push public health actors to balance the value of digital data against the rights of individuals, fostering more responsible and defensible uses of social media information [78,79,82].

National policies on digital health data are shaping how social media information can be used for surveillance, research, and public health communication [78,82,83]. These policies often build on general data protection laws but add specific rules for health‑related applications, such as requirements for ethical review, data localisation, and mandatory reporting of breaches [78,82,83]. In some countries, public health authorities have been granted exemptions to collect and analyse social media data during emergencies, while others maintain strict controls even in crises [78,82,83]. This variation creates a patchwork of rules that researchers must navigate when working across borders or with global platforms [79,83,84]. Studies of national approaches highlight both strengths and gaps [78,82,83]. Where policies are clear and well‑enforced, they can enable innovative uses of digital data while protecting privacy, but vague or overly restrictive rules can hinder timely surveillance and research [78,82,83]. For example, some nations require pre‑approval for any health‑related data collection from social media, while others leave it to platform discretion, creating uncertainty for public health teams [78,82,83]. Effective national policies therefore need to strike a balance: providing legal clarity and flexibility for legitimate public‑health needs while safeguarding against misuse, commercial exploitation, or surveillance creep [78,82,83].

International organisations such as the World Health Organization (WHO) and the European Centre for Disease Prevention and Control (ECDC) play an important role in shaping global norms for social media data use in public health [79,82,84]. These bodies develop guidelines, best‑practice frameworks, and recommendations that help countries harmonise their approaches to digital surveillance while addressing cross‑border challenges like data flows and privacy standards [79,82,84]. By convening experts and stakeholders, they also facilitate knowledge sharing about what works and what does not in using social media data responsibly [79,82,84]. For example, WHO has issued principles for ethical digital epidemiology, emphasising consent, transparency, and proportionality, while ECDC has supported pilot projects that test surveillance methods across European borders [79,82,84]. These efforts help fill gaps in national regulation and create a baseline for accountability that platforms and researchers can reference [79,82,84]. However, international organisations lack direct enforcement power, so their influence depends on voluntary adoption by member states and private companies [79,82,84]. As digital health threats grow more transnational, the coordinating role of these bodies will become even more critical for ensuring that social media data serves public health without compromising individual rights [79,82,84].

Legal risks for misusing social media data in public health contexts can be substantial and multifaceted, affecting researchers, institutions, and platforms alike [78,82,83]. The most immediate risks involve violations of data protection laws, such as collecting personal information without consent, failing to anonymise data adequately, or using it for purposes beyond what was originally authorised [78,82,83]. Such breaches can result in fines, lawsuits, and reputational damage, particularly when identifiable health data is involved [78,82,83]. Other risks stem from failing to obtain ethical approval, breaching platform terms of service, or inadvertently discriminating against protected groups through biased data practices [78,82,83]. In some jurisdictions, public health actors have faced challenges when surveillance efforts were perceived as disproportionate or discriminatory, leading to public backlash and legal scrutiny [78,82,83]. To mitigate these risks, studies recommend rigorous legal review, clear documentation of data use, and ongoing consultation with privacy experts [79,83,84]. While the potential of social media data for public health is significant, ignoring legal risks can undermine trust, halt projects, and set back legitimate research, making compliance not just a legal obligation but a practical necessity [78,82,83].

Cross‑border data governance poses significant challenges for using social media data in global public health surveillance [79,82,84]. Different countries have varying data protection standards, consent requirements, and definitions of personal information, making it difficult to move data across borders without violating one jurisdiction's laws [79,82,84]. For example, what counts as anonymised data in one country may still be identifiable under another country's stricter rules, creating legal uncertainty for multinational research teams [79,82,84]. Platforms operating globally must also comply with the most stringent regulations, which can limit data sharing even when public health benefits are clear [79,82,84]. These challenges are particularly acute during pandemics, when timely information flow is essential but legal barriers slow collaboration [79,82,84]. Studies have called for harmonised international standards, mutual recognition agreements, and federated data systems that allow analysis without physical data transfer [79,82,84]. Until such solutions are in place, cross‑border governance will remain a bottleneck for digital epidemiology, requiring public health actors to invest in legal expertise, build trust across jurisdictions, and explore technical workarounds that respect diverse regulatory environments [79,82,84].

Challenges and limitations of using social media in public health practice

Data Quality, Bias, and Representativeness Issues

Social media data for public health analysis often over‑represents certain demographic groups, creating bias that can distort disease surveillance and population estimates [2,33,50]. Younger people, urban residents, and more educated or affluent individuals tend to post more frequently and openly about health concerns, while older adults, rural populations, and lower‑income groups are under‑represented or absent [2,33,50]. This imbalance means that signals extracted from social media may reflect the experiences and concerns of digitally active subgroups rather than the population as a whole [2,33,50]. For example, symptom trends or vaccination sentiment derived from platforms like Twitter may capture urban youth perspectives but miss how rural elders or offline communities perceive the same issues [2,33,50]. Researchers have noted that this skew can lead to over‑estimation of awareness or compliance in privileged groups and under‑estimation of barriers faced by marginalised ones [2,33,50]. Adjusting for these biases is difficult because demographic details are often incomplete or self‑reported inaccurately, and simple weighting schemes may not fully correct for non‑participation [2,33,50]. The result is surveillance data that are useful for some purposes but unreliable for generalising to national or global trends, highlighting the need to supplement social media analysis with representative surveys or administrative records to ensure equitable and accurate public‑health insights [2,33,50].

The digital divide limits the representativeness of social media data for public health, because not everyone has equal access to platforms or the means to participate actively [2,6,52]. Older people, low‑income households, rural residents, and those in developing regions are less likely to use social media, less comfortable posting about health, or completely offline [2,6,52]. This exclusion means that surveillance signals drawn from digital platforms primarily reflect the views and experiences of connected, younger, urban populations, leaving out large segments of society [2,6,52]. In practice, this divide can lead to misguided public‑health responses, such as assuming high online awareness equals real‑world behaviour change, when offline groups face different barriers and information sources [2,6,52]. Studies have shown that digital surveillance underestimates disease burden or hesitancy in under‑connected areas, potentially directing resources away from where they are most needed [2,6,52]. Bridging the gap requires hybrid approaches that combine social media with traditional methods like phone surveys, community health workers, or paper‑based reporting, but these add complexity and cost [2,6,52]. Until access becomes more universal, the digital divide remains a fundamental limitation, reminding analysts that social media data offer only a partial, privileged window into public health realities rather than a complete picture [2,6,52].

Bots and fake accounts undermine the quality of social media data for public health by injecting artificial signals that distort genuine patterns of disease discussion and sentiment [64,70,72]. Automated accounts can flood conversations with repetitive claims, hashtags, or reactions, making it appear that particular health narratives have more support than they actually do [64,70,72]. These synthetic contributions are especially problematic for surveillance, because they inflate or suppress indicators of awareness, concern, or compliance, leading to flawed outbreak predictions or intervention targeting [64,70,72]. Detecting bots is challenging, as sophisticated ones mimic human language, timing, and networks, evading simple filters [64,70,72]. In some cases, coordinated campaigns have used thousands of fake profiles to amplify misinformation about vaccines or treatments, confusing analysts and the public alike [64,70,72]. Researchers have developed machine‑learning classifiers to identify suspicious activity, but these tools are imperfect and require constant updating as bot tactics evolve [64,70,72]. The presence of fakes therefore forces public health teams to invest in data cleaning, validation, and cross‑checking with reliable sources, adding burden and uncertainty to what is already an approximate method of monitoring [64,70,72]. Without better platform‑level controls, bots will continue to compromise the integrity of social media as a data source for health surveillance [64,70,72].

Language and cultural biases in social media data can skew public health analysis, because platforms and algorithms often prioritise dominant languages and Western cultural contexts [33,63,66]. Content in English, Spanish, or Mandarin receives more attention and processing resources, while posts in minority languages or dialects are under‑detected or poorly translated, missing important health signals from diverse communities [33,63,66]. Cultural differences in how illness is described-whether through metaphors, taboos, or local terms-further complicate automated analysis, as models trained on one cultural context may misinterpret or ignore expressions from others [33,63,66]. For example, symptom reports using indigenous terms or indirect phrasing may not match keyword lists designed for urban English speakers, leading to underestimation of disease activity in non‑dominant groups [33,63,66]. During global outbreaks, this bias has meant that surveillance focused disproportionately on high‑profile regions, overlooking early signs in linguistically marginalised areas [33,63,66]. Addressing these issues requires multilingual models, cultural training data, and human oversight, but such solutions are resource‑intensive and rarely implemented at scale [33,63,66]. The result is health monitoring that reflects platform demographics and algorithmic preferences more than actual population diversity, potentially exacerbating inequities in response and resource allocation [33,63,66].

Verifying the authenticity of social media posts for public health surveillance is fraught with difficulty, because self‑reported health information is often vague, exaggerated, or intentionally misleading [64,70,76]. Users may describe symptoms without medical context, use humour or metaphor, or post for attention rather than accuracy, making it hard to distinguish genuine signals from noise [64,70,76]. Bots and trolls further complicate matters by generating fake illness reports or sentiment that mimics human content but serves ulterior motives [64,70,76]. Automated verification tools struggle with nuance, context, and sarcasm, while manual checking is too slow for real‑time analysis [64, 70, 76]. Cross‑referencing with clinical data helps in some cases, but most posts cannot be matched to confirmed cases, leaving analysts to rely on probabilistic estimates that carry high uncertainty [64, 70, 76]. This verification gap means surveillance based on social media is inherently approximate, prone to false positives that waste resources or false negatives that miss emerging threats [64,70,76]. Studies emphasise the need for transparent methods, conservative interpretation, and hybrid validation using multiple sources, but the fundamental challenge of unverified posts limits the reliability of digital epidemiology for critical decision‑making [64,70,76].

Operational and Technological Constraints

Low‑resource settings face significant technical barriers to using social media for public health, including limited access to reliable internet, computing power, and skilled personnel [2,6,52]. Many health facilities lack the hardware, software, or bandwidth needed to collect, process, and analyse large volumes of digital data in real time [2,6,52]. Staff may also be stretched thin, with little time or training to monitor social media alongside clinical duties [2,6,52]. This creates a gap between the promise of digital surveillance and the reality of implementation in underfunded systems [2,6,52]. Studies from these contexts show that even when platforms are accessible, the lack of technical infrastructure often means missed opportunities for early warning or targeted communication [2,6,52]. For example, health workers might recognise the value of tracking local discussions about symptoms, but without tools for automated analysis or visualisation, the process becomes manual and unsustainable [2,6,52]. Power outages, poor connectivity, and competing priorities further compound these challenges [2,6,52]. Building capacity requires investment in basic digital infrastructure, training, and low‑cost solutions tailored to resource constraints, but funding and political will remain inconsistent [2,6,52]. Without addressing these operational hurdles, social media risks remaining an aspiration rather than a practical tool for public health in low‑resource environments [2,6,52].

Social media platforms impose data access restrictions that limit public health surveillance, creating barriers between valuable user content and those who need it most [73-75]. Application programming interfaces control what researchers can collect, often limiting volume, time range, or granularity to protect commercial interests or user privacy [73-75]. Paid access tiers exclude smaller organisations or those in low‑resource settings, while terms of service can change abruptly, breaking existing tools [73-75]. These restrictions make it difficult to monitor health trends consistently or retrospectively [73-75]. Studies have documented how platform policies have tightened over time, reducing the feasibility of large‑scale digital epidemiology [73-75]. For example, limits on historical data prevent analysis of past outbreaks, and reduced access to demographic or location information hampers targeted surveillance [73-75]. Public health agencies often lack the legal or financial leverage to negotiate better terms, leaving them dependent on platform goodwill [73-75]. Advocates call for public‑private partnerships that recognise health surveillance as a public good, but commercial priorities frequently prevail [73-75]. These access constraints underscore the vulnerability of digital public health to decisions made by private companies, highlighting the need for diversified data sources and open standards that reduce platform dependence [73-75].

The rapid evolution of social media ecosystems poses ongoing challenges for public health surveillance, as platforms, features, and user behaviours change faster than monitoring tools can adapt [10,12,21]. New apps emerge, old ones decline, algorithms shift, and user preferences migrate, requiring constant updates to data collection and analysis methods [10,12,21]. What works for Twitter today may be irrelevant on TikTok tomorrow, and privacy updates can suddenly block access to previously public content [10,12,21]. This pace disrupts long‑term trend monitoring and forces agencies to chase rather than lead [10,12,21]. Literature notes that public health teams often lack the resources to keep up, with technical staff stretched across multiple platforms and methods [10,12,21]. For example, the rise of short‑form video content demands new natural language processing and visual analysis tools, while ephemeral messaging apps challenge real‑time surveillance [10,12,21]. Cross‑platform migration further fragments data, making it harder to get a complete picture of health conversations [10,12,21]. To address this, some advocate for modular, adaptable systems and investment in flexible expertise, but the reality is that most health organisations struggle to maintain even basic monitoring [10,12,21]. The relentless evolution of digital ecosystems means public health surveillance is always playing catch‑up, risking obsolescence unless supported by sustained technical capacity and strategic foresight [10,12,21].

Integrating social media data into existing health systems presents technical, organisational, and cultural challenges that slow adoption [27,28,31]. Legacy systems designed for structured clinical data struggle to incorporate unstructured, real‑time digital streams, requiring new pipelines for ingestion, cleaning, and analysis [27,28,31]. Health workers may lack training to interpret social‑media signals alongside traditional metrics, and organisational silos can prevent data sharing between digital and clinical teams [27,28,31]. Studies highlight interoperability issues, where platform data formats change or access is restricted, breaking integration workflows [27,28,31]. Cultural resistance also plays a role, as clinicians trained in evidence‑based medicine may view social media insights as anecdotal or unreliable compared to laboratory reports [27,28,31]. Resource constraints exacerbate these problems, particularly in underfunded systems where basic functions take priority over experimental digital tools [27,28,31]. Successful integration requires cross‑disciplinary collaboration, user‑friendly interfaces, and pilot projects that demonstrate value before full‑scale rollout [27,28,31]. Without addressing these multifaceted barriers, social media data risks remaining siloed from core health systems, limiting its potential to enhance surveillance and response [27,28,31].

The sustainability of digital surveillance initiatives depends on reliable funding, technical maintenance, and organisational commitment, all of which are often lacking [40,41,45]. Pilot projects frequently show promise but struggle to scale without ongoing resources for software updates, staff training, and platform access fees [40,41,45]. When initial grants end, many initiatives lapse, leaving gaps in monitoring capacity [40,41,45]. Technical debt accumulates as platforms evolve and tools become outdated, while staff turnover erodes institutional knowledge [40,41,45]. Evaluations reveal that sustainable programmes require embedded funding streams, dedicated roles, and integration into routine workflows rather than special projects [40,41,45]. Partnerships with tech firms or governments can help, but dependence on external actors introduces risks of interruption or misalignment [40,41,45]. In low‑resource settings, the challenge is particularly acute, where even basic internet access is unreliable [40,41,45]. Long‑term success demands realistic expectations, modular designs that evolve with technology, and evidence that digital surveillance delivers measurable public‑health returns worth sustaining [40,41,45]. Without these foundations, many digital initiatives risk becoming short‑lived experiments rather than enduring components of public health practice [40,41,45].

## Conclusions

Social media is pivotal in public health, enhancing disease surveillance, fostering behaviour change, and improving information flow. It facilitates early outbreak detection and public sentiment analysis through real-time user data, often preceding traditional methods. Social media platforms enable targeted health campaigns using influencers and behavioural science to encourage practices like vaccination and screening.

While they enhance coordination during crises, they also risk spreading misinformation. Effective use of social media in public health is clear, but significant challenges, including data quality issues, misinformation, and algorithmic bias, necessitate that it complements traditional methods rather than replace them. Future strategies should focus on data validation, ethical frameworks, and digital literacy to maximise benefits and create more equitable health systems.
